# Developing a whole systems action plan promoting Dutch adolescents’ sleep health

**DOI:** 10.1186/s12966-025-01711-0

**Published:** 2025-03-17

**Authors:** Danique M. Heemskerk, Maartje M. van Stralen, Jessica T. Piotrowski, Carry M. Renders, Vincent Busch

**Affiliations:** 1 Department of Health Sciences, Faculty of Science, Amsterdam Public Health Research Institute, Vrije Universiteit Amsterdam, Van Der Boechorststraat 7, 1081 BT Amsterdam, The Netherlands; 2 Department of Healthy Living, Public Health Service (GGD), Sarphati Amsterdam, Amsterdam, The Netherlands; 3 Amsterdam School of Communication Research ASCoR, University of Amsterdam, Amsterdam, The Netherlands

**Keywords:** Adolescent, Action scales model, Causal loop diagram, Complex systems, Sleep, System approach, System dynamics, Whole system action plan, Teens, Youth

## Abstract

**Background:**

Inadequate sleep health is a public health problem among Dutch adolescents with detrimental effects on their physical and mental well-being. System approaches are increasingly being used to understand and address public health problems. Therefore, a recent study created a comprehensive Causal Loop Diagram (CLD) that integrated all relevant determinants of adolescent sleep health, underlying system dynamics and potential leverage points. Building on that, the current study aims to design a ‘whole systems action plan’ to promote sleep health of Dutch adolescents, combining systems science with a participatory approach.

**Methods:**

Five (multi)stakeholder sessions with adolescents (*N* = 40, 12–15 years), parents (*N* = 14) and professionals (*N* = 13) were organized to co-create actions addressing preselected leverage points derived from the previously mapped CLD. Subsequently, three sessions with multidisciplinary representatives of regional and national oriented (health) organizations (*N* = 27) were held using the World Café Methodology to identify intervention actions as well as potential implementers. The Action Scales Model (ASM), a tool to understand and change the system at different levels (i.e., event, structure, goal, belief) of the system, was used to create a coherent whole systems action plan.

**Results:**

The created whole systems action plan consisted of 66 (sets of) actions across different ASM levels (i.e., event, structure, goal, belief) targeting 42 leverage points across five subsystems: school environment *N* = 24; mental wellbeing *N* = 17; digital environment *N* = 9; family & home environment *N* = 9; personal system *N* = 7. Per action potential implementers were identified, which included amongst others schools and public health services. The previously mapped CLD visualizing system dynamics shaping adolescent sleep health were supplemented with how dynamics can be changed via the actions identified.

**Conclusions:**

The resulting whole systems action plan provides a subsequent step in applying a whole systems approach to understand and promote adolescent sleep health. Combining a systems approach, using the ASM, and a co-creation approach was found to be mutually reinforcing and helpful in developing a comprehensive action plan. This action plan can guide strategic planning and implementation of actions that promote systemic change. With this, it is important to ensure coherence between actions being developed and implemented to increase the potential for lasting systems change.

**Supplementary Information:**

The online version contains supplementary material available at 10.1186/s12966-025-01711-0.

## Introduction

Inadequate sleep health is a pervasive and prominent public health problem among today’s adolescents. It significantly impacts important health, social, and educational outcomes, such as a healthy weight development, emotional wellbeing, social and neurocognitive development and regulation, and academic performances [1]. Given the vital role of sleep health in adolescence, a critical period of growth and social emotional development, deteriorating sleep characteristics in adolescents raise concerns. For example, in a study among 24 European and North American countries, adolescents meeting the recommended 8–10 h of good quality sleep on school days ranged between 32 and 86% [2]. In the Netherlands, adolescent sleep health is equally worrisome with more than half of 14—17-year-olds meeting this sleep duration recommendation [3] and 24% of 12–16 year-olds adolescents rating their sleep quality as poor [4]. To target these current sleep health trends effective interventions are needed [5].

However, effectively stimulating adolescent sleep health is a complex challenge due to the dynamic interplay between biological, economic-, physical, sociocultural, and political determinants [5, 6]. Thus far, preventative interventions do not consider this complexity and instead often promote healthy adolescent sleep by focusing solely on a small set of individual determinants (e.g., psychological determinants) within one setting (e.g., school setting) most often via sleep education or mindfulness/relaxation training [7]. To achieve significant, lasting changes in sleep health however, intervention efforts need to address the coherent and dynamic complexity that is shaped by the many multi-level interacting sleep health determinants and the underlying mechanisms that shape them [6]. Therefore, rather than applying linear intervention approaches that single out a small set of determinants in isolation, changing sleep health requires a holistic, ‘whole systems approach’ (WSA) [8–10].

Whole systems approaches aim to provide a broader understanding of certain (health) outcomes or behaviors and to coherently transform the entire complex web of interacting factors that underlie it [9, 11]. Some recent WSA’s have been created in context of obesity prevention [12], yet none currently exist with regards to sleep health. Recently, Heemskerk et al. [6] provided first steps in creating a WSA by developing a causal loop diagram (CLD) to gain insight into the determinants, underlying mechanisms and broader system dynamics at play with regards to adolescent sleep health. This CLD was shaped by combining a thorough review of current research evidence with the perceptions of Dutch adolescents, parents, and a range of professionals that work closely with adolescents. The CLD revealed 6 subsystems, 23 feedback loops, and approximately 60 leverage points for action within the system to enact systems change, and thus, to potentially intervene on to promote adolescent sleep health.

The in-depth understanding of the system and potential key leverage points presented in the CLD of Heemskerk et al. [6] provided important direction for future intervention efforts. Developing a coherent set of actions, i.e., a *whole systems action plan*, that target these systems dynamics as a whole, rather than addressing isolated parts, is a relevant next step in the creation of a WSA [10, 12]. However, when identifying various leverage points and considering different ways to address them, it remains challenging to select combinations of leverage points that collectively have the greatest potential to impact and bring about system change. This is where the Action Scales Model (ASM) can serve as an asset [13]. The ASM helps to conceptualize, identify and appraise actions within complex adaptive systems. It describes four levels to understand and intervene within a system (i.e., events, structures, goals and beliefs). The deepest level of the system entails the system actors’ beliefs and driving forces that determine the system’s purpose (i.e., goals). From that, the system’s structure (i.e., the organization of the system causing events to occur) and events (i.e., observable system outcomes) emerge to achieve those system goals. Deeper levels of the system (e.g., goals and beliefs) hold more potential to achieve durable, impactful system changes, but are often more difficult to change and require more effort than changing the other levels (e.g., events, structures). To achieve system change, the ASM posits that actions could be leveraged across these four levels depending on the context. Inasmuch, applying the ASM can be a valuable next step in a WSA by providing guidance on (1) which leverage points to focus upon (i.e., targeting different system levels and mechanisms) and (2) how to ensure that a coherent set of reinforcing intervention efforts emerge which are most promising to actually bring system changes. Even more so, using the ASM *with* the target population (i.e., via co-creation approach) is thought to be a useful combination to yield appropriate, effective, impactful and sustainable solutions [13, 14].

With this in mind, to contribute to the broader literature on how to apply a WSA to address a complex public health problem and to specifically contribute to the field of adolescent sleep health, the aim of this study is to develop a ‘whole systems action plan’ to promote sleep health among Dutch adolescents using co-creation with relevant stakeholders and reliance on the Action Scales Model. In doing so, both the complexity of the system of factors that shape sleep health are considered while also being tailored to the needs of its end-users and implementers.

## Methodology

### Design

**Fig. 1:**
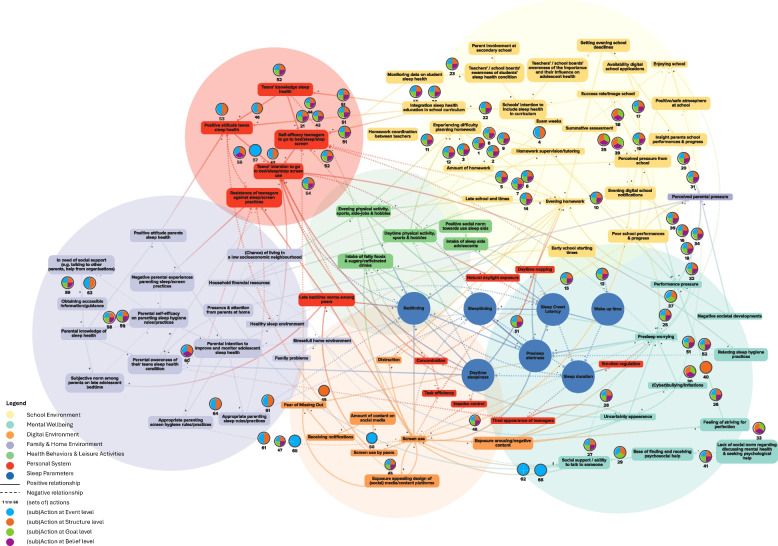
Causal loop diagram of adolescent sleep health, including all potential whole system action plan actions (adapted figure from Heemskerk et al. [6]

In this study, we build upon a previously developed CLD that was rigorously constructed using both literature and empirical data from all key stakeholders involved. This CLD aimed to capture the complex interplay of factors shaping adolescent sleep health and to identify leverage points for intervention. Thereby, this CLD served as a solid foundation for our current research. (see the CLD in Fig. 1: CLD reported by Heemskerk et al. [6]). To design a whole systems action plan, this study consisted of two phases. The first phase focused on identifying actions to impact the identified leverage points and enact the required systems changes, and thereafter in the second phase (potential) implementers and implementation strategies of said actions were identified.

#### Co-Creation, action design, phase 1

Participatory co-creation sessions were held to identify actions. Co-creation is a widely used participatory method in health research involving collaboration between academics alongside other actors (e.g., end-users of interventions and implementers) in various phases of the development of health promotion programs instead of designing these programs using a top-down approach [14]. The term actor refers to a type of stakeholder (e.g., adolescent, parent or professional), while participant refers specifically to individuals from a certain actor group that participated in the study. Co-creation yields context-sensitive and knowledge-based practices that address the complexity of a health problem and the needs of the target group. As a result, this tailoring ensures that health promotion programs are more appropriate, suitable and well-received by their target audiences, which ultimately increases the likelihood of their long-term sustainment and effectiveness [15].

**Fig. 2:**
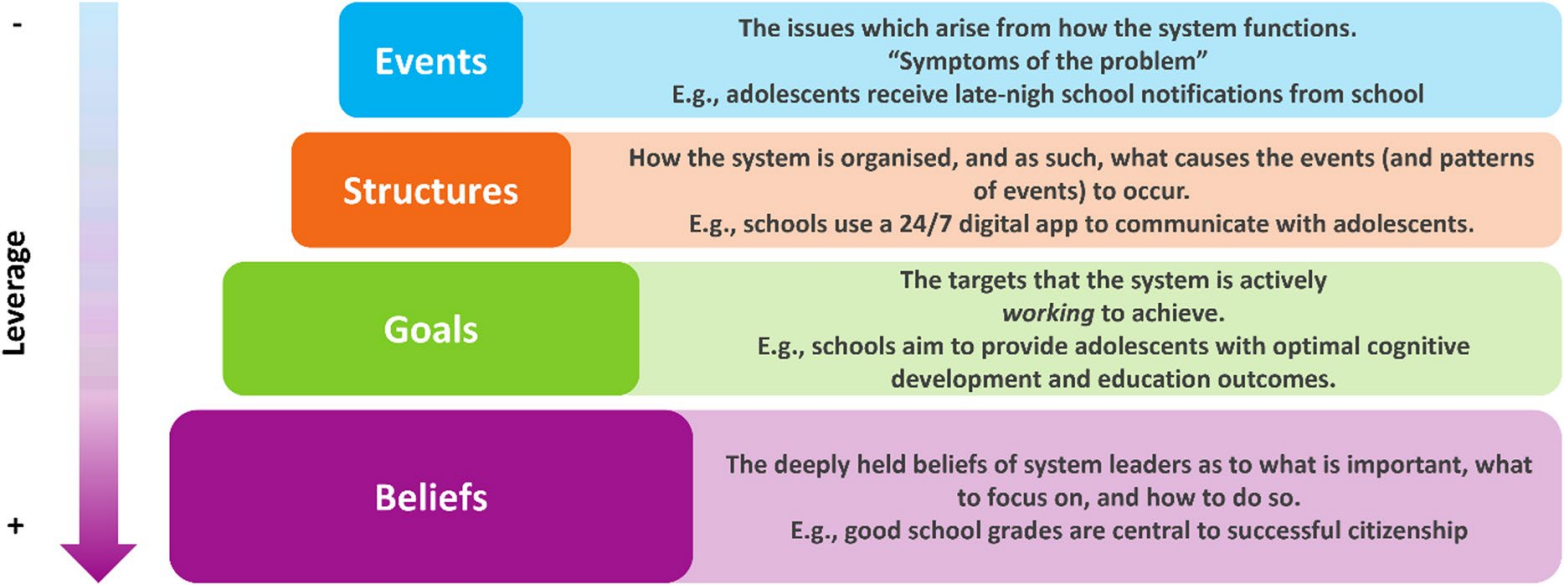
The action scales model levels* and examples** – adapted from Nobles et al. [13] *Figure and explanations ASM levels adapted from: Nobles et al. [13]. **Operationalization of ASM concepts derived from heemskerk et al. [6]

To ensure that the identified actions were not only fitting but also had an optimal potential for sustained systems change, the Action Scales Model (ASM) was applied to ensure that actions were developed across relevant system levels for each selected leverage point of this study. The ASM describes four interconnected system levels (i.e., events, structures, goals and beliefs) that facilitate understanding and intervening within a system to enact change. Understanding the system according to the ASM was part of the previous study [6]. To achieve system change action across different levels is required, whereby deeper levels (e.g., goals and beliefs) have more potential. However, these deeper levels are harder to change and demand more effort compared to the more surface levels (e.g., events and structures) [13]. Figure 2 illustrates the ASM levels and offers specific examples on how you can operationalize the levels.

#### Co-Creation, identifying implementers, phase 2

Using the insights of the first phase, co-creation sessions using the ‘World Café’ methodology was conducted for phase 2 of the project. This is a structured and participatory co-creation method used to facilitate open, meaningful, and collaborative discussions [16]. This phase was held to identify potential missed actions (e.g., additional actions and actions on deeper system levels), and to identify actor(s) and/or organization(s) that could potentially implement these actions.

### Participants and recruitment

For phase 1, relevant actors in five identified subsystems of Heemskerk et al. [6] were invited to participate. The main relevant actor groups included adolescents (aged 12–15 years) with prevocational secondary education (in Dutch; VMBO)^1^ [17], since this group report the poorest sleep health among Dutch adolescents [4]; parents or caregivers of an adolescent; and youth-, health-, digital technology and educational professionals, who were purposefully sampled based on the previous identified leverage points (by Heemskerk et al. [6]). Adolescents were recruited via a youth panel, via schools, and via social media channels. Parents (or caregivers) did not have to be the parents of the adolescents participated in the study, but rather any parent of an adolescent in general aged 12–15 years. They were recruited via different social media platforms and with help of professionals from the different participating Public Health Services. Professionals were recruited via a network of affiliated municipalities and Public Health Services of the project. We aimed to include 6–12 participants per co-creation session [14]. Participants received a gift card of 25 euros for participation per workshop.

In phase 2, the ‘World Café Session’, relevant actors at both local and national level who might be responsible for implementing and supporting the identified actions from phase 1 were recruited. These participants were recruited using the network of affiliated municipalities, Public Health Services, and practice-based organizations that had multidisciplinary backgrounds and/or worked at different (municipal) sectors (e.g., health promotion workers/public health advisors, youth healthcare professionals, policy advisors public (mental) health).

A total of 40 adolescents from pre-vocational secondary education (aged 12–15 years, mean age 14 years, 20 girls), 14 parents (11 female), and 40 professionals, 13 in phase 1 (e.g., teachers, sport coaches, mental health experts, digital health experts, parenting expert) and 27 in phase 2 (e.g., Healthy School Advisors, health promotion workers/public health advisors, parent–child advisors, youth psychologists/doctors, youth healthcare professionals, policy advisors public (mental) health, policy advisors healthy living environment, policy advisors youth and digital environment) participated in 8 multi-actor and single-actor co-creation sessions with attendance ranging from 9–18 participants per session. During the sessions with adolescents, we ensured that they formed the majority of participants and that all participants experienced the sessions as a safe space and open environment in which they were comfortable to share experiences and thoughts. All participants received information about the co-creation session prior to participation and provided active informed consent. With regards to the participating adolescents, their parents or caregivers provided passive informed consent.

Prior to study onset, ethical approval was granted by the institutional medical ethics committee of Amsterdam UMC (VUMC 2021.0783).

### Procedure

#### Co-creation phase 1: Action design

Between April and June 2022, five co-creation sessions were conducted with the aim of identifying and co-creating program actions at the selected leverage points. Each session focused on a specific adolescent sleep health *subsystem* (i.e., school environment, mental wellbeing, digital environment, personal system, and family & home environment) [6] and lasted 2 h. Depending on relevance and content, we held either multi-actor sessions (e.g., school environment, mental wellbeing, digital environment) or single-actor sessions (e.g., family & home environment and personal system).

In the previous study of Heemskerk et al. [6], about sixty potential leverage points were identified. Investigating all of these was not feasible for the co-creation sessions. For that reason, the number of leverage points that were addressed in the creation of the action plan were reduced. First, some leverage points were combined because they essentially addressed the same points or complemented each other. Next, a prioritization was made by the research team resulting in 15 leverage points (see Table 1, Result section) based on their changeability and potential impact within the system. This selection was informed by evidence from the scientific literature and consultation with experts (i.e., their influence to strengthen or break a feedback loop or mechanism).

**Table 1:** Adolescent sleep health whole systems action plan

School environment					
**Mechanism**	**Leverage Point**	**Action Number**	**Actions**	**ASM Level**	**Potential Implementers**
Evening Homework and Planning	**LP1:** Reducing evening homework (Event) **LP2:** Aiding adolescents in planning their homework (Event)	1	Teachers avoid assigning homework for the following day	Event	Teachers
			Schools have a policy stating to assign little to no homework for the next day	Structure	School board
			Schools set the explicit organisational aim to facilitate students to have sufficient leisure time after school hours	Goal	School board
			Raise awareness among schools that their students have the right to leisure time after school hours to keep a healthy school-private life balance and protect their mental wellbeing	Belief	Local public health services' (in Dutch: GGD) healthy school advisors; the national Dutch Healthy School Program; [18] Youth at a healthy weight program (in Dutch: JOGG); [19] the Dutch Council for Secondary Education (in Dutch: VO Raad); [20] the Dutch Youth Institute (in Dutch: Nji); [21] the Dutch Ministry of Education, Culture and Science; the Dutch Ministry of Health, Social welfare, and Sports
		2	Students get their homework details communicated in time, at the latest at 4:00 PM	Event	Teachers
			School have a policy stating that homework details are communicated in time, at the latest at 4:00 PM	Structure	School board
			Schools set the explicit organisational aim to facilitate students to have sufficient leisure time after school hours	Goal	School board
			Raise awareness among schools that their students have the right to leisure time after school hours to keep a healthy school-private life balance and protect their mental wellbeing	Belief	Local public health services' healthy school advisors; the national Dutch Healthy School Program; JOGG; the Dutch Council for Secondary Education; the Dutch Youth Institute; the Dutch Ministry of Education, Culture and Science; the Dutch Ministry of Health, Social welfare, and Sports
		3	Students receive a maximum amount of homework of two hours a day	Event	Teachers
			Schools have a policy that states that homework assignments are limited to a maximum of two hours a day	Structure	School board
			Schools set the explicit organisational aim to facilitate students to have sufficient leisure time after school hours	Goal	School board
			Raise awareness among schools that their students have the right to leisure time after school hours to keep a healthy school-private life balance and protect their mental wellbeing	Belief	Local public health services' healthy school advisors; the national Dutch Healthy School Program; JOGG; the Dutch Council for Secondary Education; the Dutch Youth Institute; the Dutch Ministry of Education, Culture and Science; the Dutch Ministry of Health, Social welfare, and Sports
		4	Adolescents get a week off from school activities preceding an exam week for preparation	Event	School board
			Schools have a policy that states that students get a designated week off from school activities preceding exam week to provide them sufficient preparation time	Structure	School board
		5	Students get taught via the so-called "Flip the classroom method", meaning they receive a video outlining the content of the upcoming lesson, so that they come prepared to class where they work on their assignments under the supervision of a teacher	Event	Teachers; School board
			Schools implement the "flipping the classroom"	Structure	School board
			Schools set the explicit organisational aim to facilitate students to have sufficient leisure time after school hours	Goal	School board
			Raise awareness among schools that their students have the right to leisure time after school hours to keep a healthy school-private life balance and protect their mental wellbeing	Belief	Local public health services' healthy school advisors; the national Dutch Healthy School Program; JOGG; the Dutch Council for Secondary Education; the Dutch Youth Institute; the Dutch Ministry of Education, Culture and Science; the Dutch Ministry of Health, Social welfare, and Sports
		6	Adolescents have the opportunity to work on their homework at school	Event	Teachers; School board
			Schools provide a classroom or other quiet workspace to do homework after or between classes	Structure	School board
			Schools set the explicit organisational aim to facilitate students to have sufficient leisure time after school hours	Goal	School board
			Raise awareness among schools that their students have the right to leisure time after school hours to keep a healthy school-private life balance and protect their mental wellbeing	Belief	Local public health services' healthy school advisors; the national Dutch Healthy School Program, Council for Secondary Education; Dutch Ministry of Education, Culture and Science
		7	Students can start/do homework during class	Event	Teachers; School board
			Schools provide students with the opportunity, and stimulate them, to start with homework during class	Structure	School board
			Schools set the explicit organisational aim to facilitate students to do their homework at school, so students have sufficient leisure time after school hours	Goal	School board
			Raise awareness among schools that their students have the right to leisure time after school hours to keep a healthy school-private life balance and protect their mental wellbeing	Belief	Local public health services' healthy school advisors; the national Dutch Healthy School Program; JOGG; the Dutch Council for Secondary Education; the Dutch Youth Institute; the Dutch Ministry of Education, Culture and Science; the Dutch Ministry of Health, Social welfare, and Sports
		8	Students receive homework support at school	Event	Teachers
			Teachers are facilitated to provide students with opportunity to do their homework at school with their Teachers' support	Structure	School board
			Within the school curriculum more time will be allocated to organizing, planning and doing homework	Structure	Teachers; School board
			Schools set the explicit organisational aim to support students to organize, plan, and complete their homework	Goal	School board
			Raise awareness among schools that students need support with their problem-solving and self-regulatory skills, such as planning their schoolwork and daily activities	Belief	Local public health services' healthy school advisors; the national Dutch Healthy School Program; JOGG; the Dutch Council for Secondary Education; the Dutch Youth Institute; the Dutch Ministry of Education, Culture and Science
		9	Mentor, tutor (or someone else) provides assistance to adolescents in organizing/planning homework and exam weeks	Event	Teachers
			Within the school curriculum more time will be allocated to organizing, planning and doing homework	Structure	Teachers; School board
			Schools aim to provide support hours for adolescents to help them organize, plan, and complete their homework	Goal	School board
			Raise awareness among schools that students need support with their problem-solving and self-regulatory skills, such as planning their schoolwork and daily activities	Belief	Local public health services' healthy school advisors; the national Dutch Healthy School Program; JOGG; the Dutch Council for Secondary Education; the Dutch Youth Institute; the Dutch Ministry of Education, Culture and Science
	**LP3:** Prohibiting late-night school deadlines (Structure)	10	Students do not have late-night deadlines for school assignments	Event	Teachers
			Schools implement a policy that restricts late-night school deadlines	Structure	School board
			The local public health services' healthy school advisor receives tools and guidance to aid in raising awareness among school personnel and assist schools with the importance of preventing late-night deadlines	Structure	Local public health services; the national Dutch Healthy School Program
			Schools set the explicit organisational aim to facilitate students to have sufficient leisure time after school hours	Goal	School board
			Raise awareness among schools that late-night school deadlines damage student sleep health, which in turn is crucial for their cognitive development, mental wellbeing and physical health as well as damaging their right to leisure time after school hours to keep a healthy school-private life balance and protect their mental wellbeing	Belief	Local public health services' healthy school advisors; the national Dutch Healthy School Program; JOGG; the Dutch Council for Secondary Education; the Dutch Youth Institute; the Dutch Ministry of Education, Culture and Science; the Dutch Ministry of Health, Social welfare, and Sports
	**LP4:** Homework coordination between Teachers about when and how much homework they give (Structure)	11	Students do not receive an overload of workload due to simultaneous homework peaks from multiple classes	Event	Teachers
			Teachers coordinate homework assignments with other subjects to prevent workload peaks	Structure	Teachers
			Schools utilize one digital platform for communication and sharing homework assignments with adolescents to prevent unexpected workload peaks	Structure	Teachers; School board
			Schools set the explicit organisational aim to facilitate students to have sufficient leisure time after school hours	Goal	School board
			Raise awareness among schools that their students have the right to leisure time after school hours to keep a healthy school-private life balance and protect their mental wellbeing	Belief	Local public health services' healthy school advisors; the national Dutch Healthy School Program; JOGG; the Dutch Council for Secondary Education; the Dutch Youth Institute; the Dutch Ministry of Education, Culture and Science; the Dutch Ministry of Health, Social welfare, and Sports
		12	Students receive examinations evenly spread throughout each trimester instead of via extreme peak moments	Event	School board
			Schools are provided with tools and guidance on how to implement changes regarding exam timetables	Structure	Local public health services' healthy school advisors; the national Dutch Healthy School Program; Public health services
			Schools schedule no exam weeks, but rather distribute exams evenly throughout each trimester to prevent peaks in workload and subsequent stress with students	Structure	School board
			The local public health services' healthy school advisor receives tools and guidance to aid in raising awareness among school personnel and assist schools with the importance of structuring exam weeks throughout the trimester	Structure	Local public health services'; the national Dutch Healthy School Program
			Schools set the explicit organisational aim to facilitate students to have sufficient leisure time after school hours	Goal	School board
			Raise awareness among schools that their students have the right to leisure time after school hours to keep a healthy school-private life balance and protect their mental wellbeing	Belief	Local public health services' healthy school advisors; the national Dutch Healthy School Program; JOGG; the Dutch Council for Secondary Education; the Dutch Youth Institute; the Dutch Ministry of Education, Culture and Science; the Dutch Ministry of Health, Social welfare, and Sports
School timetable	**LP5:** Aligning school schedules with students’ biorhythm (e.g. starting- and end times, breaks) (Structure)	13	Students do not have classes prior to 9 AM	Event	School board
			Schools are provided with tools and guidance on how to implement changes regarding school start times	Structure	Local public health services' healthy school advisors; the national Dutch Healthy School Program; JOGG
			Schools implement a policy that states that school start times are no earlier than 9 AM	Structure	School board
			The local public health services' healthy school advisor receives tools and guidance to aid in raising awareness among school personnel and assist schools with the importance of delaying school start times	Structure	Local public health services'; the national Dutch Healthy School Program
			Schools align school schedules with students’ biorhythm	Goal	School board; Local Public Health Services, the National Dutch Healthy School program; JOGG
			Strengthen the belief among schools that sleep health is crucial for the mental health and cognitive development of adolescents	Belief	Local public health services' healthy school advisors; the national Dutch Healthy School Program; JOGG; the Dutch Youth Institute; local Municipal Departments of Health; local Municipal Department of Education; the Dutch Council for Secondary Education; the Dutch Ministry of Education, Culture and Science; the Dutch Ministry of Health, Social welfare, and Sports
			Create awareness, e.g. via lobbying, among national politicians and policy makers about the importance to better align school start times with the adolescent biorhythm	Belief	Local public health services; the Dutch Council for Secondary Education; the Dutch Youth Institute; local municipalities
		14	Students do not have classes that run later than 4:00 PM	Event	School board
			Schools are provide tools and guidance on how to implement changes regarding school end times	Structure	Local public health services' healthy school advisors; the national Dutch Healthy School Program; JOGG
			Schools implement a policy that states that school end times are no later than 4:00 PM	Structure	School board
			The local public health services' healthy school advisor receives tools and guidance to aid in raising awareness among school personnel and assist schools with the importance of appropriate school end times	Structure	Local public health services; the national Dutch Healthy School Program
			Schools align school schedules with students’ biorhythm	Goal	School board
			Raise awareness among schools that their students have the right to leisure time after school hours to keep a healthy school-private life balance and protect their mental wellbeing	Belief	Local public health services' healthy school advisors; the national Dutch Healthy School Program, the Dutch Council for Secondary Education; the Dutch Youth Institute; the Dutch Ministry of Education, Culture and Science; the Dutch Ministry of Health, Social welfare, and Sports
		15	Students do not undergo any tests prior to 11:00 AM	Event	School board
			School are provided with tools and guidance (e.g. policy templates) on how to implement changes to their exam timetables that allow for a better alignment with students’ biorhythm	Structure	Local public health services' healthy school advisors; the national Dutch Healthy School Program; JOGG; the Dutch Youth Institute
			Schools set no tests or examinations to take place before 11:00 AM	Structure	School board
			School align their schedules with students’ biorhythm	Goal	School board
			The local public health services' Healthy School Advisor receives tools and guidance to raise awareness among school personnel about the importance of delaying times of examinations to strengthen the belief that sleep health is crucial for adolescents' cognitive development, mental wellbeing and physical health	Belief	Local public health services; the national Dutch Healthy School Program; JOGG; the Dutch Youth Institute
Evening school notifications	**LP6:** No digital communication from schools in the evening (Structure)	16	Students do not receive any digital notifications from school and they cannot access the online grading and test scores system between 8:00 PM and 8:00 AM	Event	School board
			Schools implement policies that prevent sending students notifications as well as to prevent them from accessing the online test scores and grading system between 8:00 PM and 8:00 AM	Structure	School board
			Schools are provided with tools and guidance such as policy advice templates and good practices examples on how to implement restrictions related to digital communication between Teachers and adolescents in the evening	Structure	Local public health services' healthy school advisors; the national Dutch Healthy School Program; JOGG; the Dutch Youth Institute
			Schools aim to protect students' mental wellbeing and prevent stress	Goal	School board
			Schools are made aware that evening notifications (e.g. communicating test results) causes students stress that in turn negatively affects their sleep health	Belief	Local public health services' healthy school advisors; the national Dutch Healthy School Program; JOGG; the Dutch Council for Secondary Education; the Dutch Youth Institute
			Raise awareness among schools that their students have the right to leisure time after school hours to keep a healthy school-private life balance and protect their mental wellbeing	Belief	Local public health services' healthy school advisors; the national Dutch Healthy School Program, the Dutch Council for Secondary Education; the Dutch Youth Institute; the Dutch Ministry of Education, Culture and Science; the Dutch Ministry of Health, Social welfare, and Sports
		17	Schools' digital applications do not automatically publish test scores, grades, homework and schedule changes, but rather give Teachers the opportunity to do so when they choose to on preset times	Event	Digital (mobile) school application software developers
			Students do not receive communications from digital (mobile) school applications about their test scores and grades between 8 PM and 8 AM	Event	School board
			Schools only use digital (mobile) applications that allow for sending students messages at preset times	Structure	School board
			Software developers provide the opportunity to Teachers to publish grades, homework and schedule changes on a later timestamp than when Teachers initially submit them	Structure	Digital (mobile) school application software developers
			Schools aim to protect students' mental wellbeing and prevent stress	Goal	School board
			Digital (mobile) school application software developers develop digital (mobile) school applications that take into account the (mental) health consequences their product has on students	Goal	Digital (mobile) school application software developers
			Schools are made aware of the harmful effects of sending their students stressful messages in the evening	Belief	Local public health services' healthy school advisors; the national Dutch Healthy School Program; JOGG; the Dutch Council for Secondary Education; the Dutch Youth Institute
		18	Teachers do not specify the exact day and time that they will publish students' test scores and grade	Event	Teachers
			Schools implement a policy that prohibits teachers from specifying when test grades will be published	Structure	School board
			Schools aim to protect students' mental wellbeing and prevent stress	Goal	School board
			Teachers are made aware about the effect of specifying the exact day and timing of grade publication on sleep health to strengthen the belief that this anticipation causes stress and in turn affects sleep health	Belief	Local public health services' healthy school advisors; the national Dutch Healthy School Program; JOGG; the Dutch Council for Secondary Education; the Dutch Youth Institute
			Raise awareness among schools that their students have the right to leisure time after school hours to keep a healthy school-private life balance and protect their mental wellbeing	Belief	Local public health services' healthy school advisors; the national Dutch Healthy School Program; JOGG; the Dutch Council for Secondary Education; the Dutch Youth Institute; the Dutch Ministry of Education, Culture and Science; the Dutch Ministry of Health, Social welfare, and Sports
		19	Students can only access their test scores and grades on digital (mobile) school applications between 8:00 AM and 8:00 PM	Event	School board
			Schools set their digital (mobile) applications to limit the accessibility of students to the online grading and test scores system between 8:00 PM and 8:00 AM	Structure	School board
			Schools protect students' mental wellbeing and prevent stress by not communicating with them in the evenings or on weekends	Goal	School board
			Raise awareness among schools that their students have the right to leisure time after school hours to keep a healthy school-private life balance and protect their mental wellbeing	Belief	Local public health services' healthy school advisors; the national Dutch Healthy School Program; JOGG; the Dutch Council for Secondary Education; the Dutch Youth Institute; the Dutch Ministry of Education, Culture and Science; the Dutch Ministry of Health, Social welfare, and Sports
		20	Parents' access to students' test scores, grades, homework, and schedules via digital (mobile) school applications is restricted or limited in order to prevent stressful discussions between parent and child in the evening	Event	School board
			Schools set their digital (mobile) applications provide delayed parental access to published test scores and grades	Structure	School board
			Schools protect their students' wellbeing by limiting or restricting the digital access of parents to students' grades, homework, and schedules via school apps	Goal	School board
			Schools are made aware of the negative effects of parental real-time access to students' grades, test scores, homework assignments, and class schedules via school applications leads to stressful discussions at home and subsequently to poorer sleep and mental health	Belief	Local public health services' healthy school advisors; the national Dutch Healthy School Program; JOGG; the Dutch Council for Secondary Education; the Dutch Youth Institute; the Dutch Ministry of Education, Culture and Science; the Dutch Ministry of Health, Social welfare, and Sports
		21	Students are taught media literacy and the healthy handling of their (social) media devices in school	Event	Teachers
			Schools include (social) media literacy within their curricula	Structure	School board
			Schools strive for their students to be media literate	Goal	School board
			Schools are made aware about the importance of teaching adolescents to turn off their school notifications in the evening (e.g., sharing research results) to strengthen the belief that evening notifications increases evening stress that in turn negatively influences sleep health	Belief	Local public health services' healthy school advisors; the national Dutch Healthy School Program; JOGG; the Dutch Council for Secondary Education; the Dutch Youth Institute; the Dutch Ministry of Education, Culture and Science; the Dutch Ministry of Health, Social welfare, and Sports; local municipal departments of health; local municipal departments of education
Other	**LP7:** Include sleep health as part of the school curriculum (Structure) **LP8:** Schools are there to facilitate learning, getting good grades (do not yet play a multifaceted role in promoting health among adolescents) (current Belief)	22	Students get taught about sleep health in school with the aim to stimulate their healthy sleep habits	Event	Teachers
			Schools integrate sleep health intervention efforts into their curriculum	Structure	School board
			The Dutch National Healthy School program actively stimulates schools to integrate sleep as a healthy school theme into schools' health-promoting school efforts	Structure	the Dutch national Healthy School program
			Schools actively protect and stimulate the sleep health of their students	Goal	School board
			Schools prioritize overall well-being alongside academic achievement	Goal	School board
			Improving the sleep health of adolescents is part of the Dutch National Healthy School Program’s aims	Goal	the Dutch national Healthy School program
			Raise awareness among schools that adolescent sleep health is crucial to healthy adolescent development, cognitive functioning and learning	Belief	the Dutch Council for Secondary Education, the Dutch Ministry of Education, Culture and Science; the Dutch Ministry of public health, social welfare, science and sports; local Municipal departments of health; local Municipal departments of education
	**LP9:** Monitoring data on adolescent sleep health (Structure) **LP10:** Lack of Teachers’/school board awareness of students sleep health (or mental health) condition (current Belief)	23	Teachers and school board are periodically informed about their students' sleep health	Event	Local public health services' healthy school advisors; the Dutch National Youth Monitor
			Sleep health is included in routine adolescent health surveys	Structure	The National Dutch Youth Monitor; Local Public Health Services
			The monitoring adolescent sleep health is included in the standard governmental periodical health surveys	Goal	Collaborative local public health services (in Dutch: GGD/GGD GHOR); [22] National Institute for Public Health and the Environment (in Dutch: RIVM) [23]
			Raising awareness that monitoring adolescent sleep health is seen as a key aspect of monitoring overall health and wellbeing	Belief	Local and collaborative public health services; the national Dutch Healthy School Program; the Dutch Council for Secondary Education; the Dutch Youth Institute
	**LP11:** Providing students with optimal cognitive, educational outcomes (not promoting adolescent (sleep) health) (current Goal)	24	Adolescents are educated about sleep health	Event	Teachers
			Schools implement sleep education in their school curriculum	Structure	School board
			Schools prioritize overall well-being alongside academic achievement	Goal	School board
			Raise awareness with School board and Teachers about the importance of, and their influence on, adolescent sleep habits on their health, wellbeing and cognitive development	Belief	Local public health services' healthy school advisors; the Dutch national Healthy School program; JOGG; the Dutch Council for Secondary Education; the Dutch Youth Institute; the Dutch Ministry of Education, Culture and Science; the Dutch Ministry of Health, Social welfare, and Sports
**Mental wellbeing**					
**Mechanism**	**Leverage Point**	**Action Number**	**Actions**	**ASM Level**	**Potential Implementers**
(coping with) Perceived performance pressure adolescents	**LP12**: Providing adolescents with knowledge, awareness, skills and tools to cope with stress & presleep worrying (e.g., relaxing sleep hygiene practices) (Event)	25	Adolescents are taught to manage and cope with stress and performance pressure in benefit of their mental wellbeing	Event	Teachers, school healthcare professionals
			Influencers, role models and peers share their experiences with adolescents about dealing with performance pressures and mental health issues, offering insights into coping mechanisms and effective strategies, preferably through online platforms	Structure	Social media influencers, youth social workers
			Schools include the topics of stress and performance pressure within the school curriculum	Structure	School board
			Schools set concrete goals to protect students' mental wellbeing and prevent stress	Goal	School board
			Raise awareness among schools about the effects of stress and performance pressure on students' mental wellbeing and on their cognitive performance e.g., using mental- and sleep health monitoring data)	Belief	Local public health services' healthy school advisors; the national Dutch Healthy School Program; JOGG; the Dutch Council for Secondary Education; the Dutch Youth Institute; Trimbos Institute; [24] the Dutch Ministry of Education, Culture and Science; the Dutch Ministry of Health, Social welfare, and Sports; local municipal departments of education and of health
	**LP13:** Providing adolescents with awareness and skills to cope with people portraying a ‘perfect life’ online. (Event)	26	Raise awareness among adolescents about the portrayed 'perfect picture' on social media versus the reality of everyday life	Event	Teachers, school healthcare professionals
			Mass media campaigns are being developed to highlight the deceptions of social media	Structure	Trimbos Institute; the Dutch Ministry of Health, Social welfare, and Sports; local municipal departments of education and of health; Netherlands Youth Institute
			Schools include media literacy within the school curriculum	Structure	School board
			Schools set concrete goals to improve the media literacy of adolescents	Goal	School board
			Raising awareness among adolescents, parents and schools that online content provides adolescents with unrealistic expectations of real life, which is of significant influence on their stress levels and damages their mental wellbeing	Belief	Local public health services' healthy school advisors; the national Dutch Healthy School Program; JOGG; the Dutch Council for Secondary Education; the Dutch Youth Institute; Trimbos Institute; the Dutch Ministry of Education, Culture and Science; the Dutch Ministry of Health, Social welfare, and Sports; municipal departments of health and education, social media influencers
	**LP14:** Providing more opportunities to talk about mental health (e.g., at school) (Structure) **LP15:** Mental health is included in the school curriculum (Structure)	27	Incorporate discussions with adolescents about stress and pressure into the classroom	Event	Teachers; care coordinator school, mental health professionals; youth social workers
			Schools include the topics of stress and performance pressure within the school curriculum	Structure	School board
			Schools set concrete goals to protect students' mental wellbeing and prevent stress	Goal	School board
			Raise awareness among schools about the effects of stress and performance pressure on students' mental wellbeing and on their cognitive performance e.g., using mental- and sleep health monitoring data)	Belief	Local public health services' healthy school advisors; the national Dutch Healthy School Program; JOGG; the Dutch Council for Secondary Education; the Dutch Youth Institute; Trimbos Institute; the Dutch Ministry of Education, Culture and Science; the Dutch Ministry of Health, Social welfare, and Sports; local municipal departments of education and of health
		28	Offer individual counselling services for adolescents for mental health support at school	Event	School professionals (in Dutch: zorgcoordinator); school healthcare professionals, i.e. school doctors / nurses (in Dutch: Jeugdgezondheidszorgprofessionals)
			Integrate a focus on discussing students' mental well-being during the regular periodic sessions they have with the school's healthcare professional from the local public health service	Structure	School healthcare professionals
			Schools and school health care professionals concretely strive to protect and stimulate adolescents' mental wellbeing	Goal	School board; local public health services
			Raise awareness among schools and local public health services about the importance, and impact their professionals' have, on adolescent mental health	Belief	The national Dutch Healthy School Program; JOGG; the Dutch Council for Secondary Education; the Dutch Youth Institute; Trimbos Institute; the Dutch Ministry of Education, Culture and Science; the Dutch Ministry of Health, Social welfare, and Sports
		29	Adolescents can more easily find and receive psychosocial help with shorter waiting lists	Event	-
			Improve access to counselling services for mental wellbeing	Structure	Local municipal departments of education and of health
			Establish a consultation office specifically designed for adolescents (now limited contact at this age range)	Structure	Local public health services
			Society strive to protect and stimulate adolescents' mental wellbeing	Goal	Local public health services; the Dutch Ministry of health, social welfare, and sports
	**LP16:** More attention for (cyber)bullying (Event)	30	Implement existing evidence-based programs to prevent (cyber)bullying	Structure	School board
			Schools are stimulated and equipped to prevent (cyber)bullying and creating a safe school environment	Structure	Local public health services' healthy school advisors
			Schools integrate the topic of (cyber)bullying prevention and creating a safe school environment as part of their core organisational aims	Goal	School board
			Raising awareness among schools that a safe school climate is crucial for students' cognitive performance, ability to learn and for their healthy socio-emotional development	Belief	Local public health services' healthy school advisors; the national Dutch Healthy School Program; JOGG; the Dutch Council for Secondary Education; the Dutch Youth Institute; Trimbos Institute; the Dutch Ministry of Education, Culture and Science; the Dutch Ministry of Health, Social welfare, and Sports; municipal departments of health and education
	**LP17**: No digital communication (e.g., about homework, grades, schedule changes) from schools in the evening (Structure)	31	Individually communicate grades to adolescents before publishing them online to reduce stress and provide an opportunity for teachers to offer support and reassurance	Event	Teachers
			Schools implement a policy/regulation that grades must be personally communicated to teenagers before publishing them online	Structure	School board
			Schools aim to protect students' mental wellbeing, prevent stress and respect students' autonomy and privacy	Goal	School board
			Schools are made aware that evening notifications (e.g. communicating test results) causes students stress that in turn negatively affects their sleep health	Belief	Local public health services' healthy school advisors; the national Dutch Healthy School Program; JOGG; the Dutch Council for Secondary Education; the Dutch Youth Institute
			Raise awareness among schools that their students have the right to leisure time after school hours to keep a healthy school-private life balance and protect their mental wellbeing	Belief	Local public health services' healthy school advisors; the national Dutch Healthy School Program, the Dutch Council for Secondary Education; the Dutch Youth Institute; the Dutch Ministry of Education, Culture and Science; the Dutch Ministry of Health, Social welfare, and Sports
		32	Provide timely communication to adolescents regarding any adjustments to the school schedule (not in the evening to prevent evening stress)	Event	Teachers; scheduler
			Schools implement policy/regulations and establish agreements with teachers regarding sending evening school notifications and visibility of the online grading system in the evening, e.g., • Teachers notify students about homework or grades by a specific time (e.g., by 6:00 PM) • No school notifications after a designated time, for instance, 8:00 PM	Structure	Teachers; School board
			Schools set the explicit organisational aim to facilitate students to have sufficient leisure time after school hours	Goal	School board
			Raise awareness among schools that sending evening school notifications damage student sleep health, which in turn is crucial for their cognitive development, mental wellbeing and physical health as well as damaging their right to leisure time after school hours to keep a healthy school-private life balance and protect their mental wellbeing	Belief	Local public health services' healthy school advisors; the national Dutch Healthy School Program; JOGG; the Dutch Council for Secondary Education; the Dutch Youth Institute; the Dutch Ministry of Education, Culture and Science; the Dutch Ministry of Health, Social welfare, and Sports
		16	See action #16 within ‘school environment’		
		19	See action #19 within ‘school environment’		
	**LP18:**Adolescents: complying with the social norm (e.g., not talking about stress and mental wellbeing) and getting recognition from peers (current Goal)	33	Facilitate open discussions among adolescents to share their experiences with stress, performance pressures and mental wellbeing (via e.g., peer-to-peer-education)	Structure	School board; Teachers; youth (school) healthcare professionals; youth workers
			Encourage influencers, role models and peers to share their experiences with adolescents dealing with performance pressures and mental health issues, offering insights into coping mechanisms and effective strategies, preferably through online platforms	Structure	Social media influencers; youth social workers
			To foster an environment where adolescents feel comfortable and supported to share their struggles with stress, performance pressure and mental wellbeing	Goal	School board
			Raise awareness among adolescents about the importance of (mental) health and the influence of their peers on their behavior	Belief	Schools; youth workers; (social media) influencers
Perceived parental pressure	**LP19:** Schools limit their digital communication structures with adolescents and parents (Structure)	11	See action #11 within ‘school environment’		
	**LP20:** Perceived pressure from parents: limiting/restricting parental digital access to school performances (Structure)	20	See action #20 within ‘school environment’		
	**LP21:** Parents: ensuring the best possible future for their child (current Goal)	34	Provide parents with information on (how to address) their child(ren)'s performance pressure and mental wellbeing (e.g., effective methods for discussing grades with their children in a supportive manner)	Event	Local public health services' healthy school advisors and youth healthcare professionals; the national Dutch Healthy School Program; school professionals
			Structural implement e.g., host themed parent evenings at school to strengthen parental support, with a focus on addressing concerns such as performance pressures and mental health issues related to their children and providing tips & tricks	Structure	School board, Local public health services' healthy school advisors; the national Dutch Healthy School Program
			Equip parents of adolescents to effectively and supportively take on the task of supporting their child(ren) in the context of performance pressure, stress and mental wellbeing	Goal	Local public health services' healthy school advisors and youth healthcare professionals; the national Dutch Healthy School Program; school professionals
			Adolescents need to be supported by their parents on the issues of performance pressure and mental wellbeing	Belief	Trimbos Institute; Local public health services; the Dutch National Healthy School program; the Dutch Youth Institute, the Dutch Council for Secondary Education
Performance culture society (e.g., school, societal norms)	**LP22:** Schools do not focus solely on summative assessment (Structure)	35	Schools limit the amount of tests and examinations	Structure	School board
			Schools value other types of performance assessments than only tests and examinations	Structure	School board
			Schools are provided with tools and guidance, such as policy advice templates and good practices examples on how to transition away from a grade-centric approach and adopt alternative assessments to assess adolescents mastery of knowledge and focus on personal development	Structure	The Dutch Ministry of Education, Culture and Science; the local municipal department of education
			Schools should broaden their goal in stimulating adolescent development beyond end exam scores and tests	Goal	School board; the Dutch Ministry of Education, Culture and Science
			The Dutch Ministry of Education, Culture and Science is made aware of the negative consequences of solely focusing on exams on adolescents' performance pressure and mental wellbeing	Belief	Trimbos Insitute; the Dutch Youth Institute, the Dutch Council for Secondary Education
	**LP23:** Requirements/standards for further education are not based on summative assessment of cognitive development only (Structure)	36	Students have more time to develop themselves before being assigned to a specific education level	Event	School board
			Extend and expand the freshman year to allow for a more thorough assessment of students' aptitude for different learning streams and defer the determination of academic level and school choice to a later stage	Structure	Ministry of Education, Culture and Science, Council for Secondary Education
			Schools should broaden their goal in stimulating adolescent development beyond end exam scores and tests	Goal	School board; the Dutch Ministry of Education, Culture and Science
			The Dutch Ministry of Education, Culture and Science is made aware of the negative consequences of solely focusing on exams on adolescents' performance pressure and mental wellbeing	Belief	Trimbos Insitute; the Dutch Youth Institute, the Dutch Council for Secondary Education
	**LP24**: Society: being successful (i.e. dependent one’s education, carrier, income, housing, appearance and social circle) (current Goal)	37	Increase appreciation for vocational education and individuals with practical skills	Event	Influencers; Ministry of Education, Culture and Science, Council for Secondary Education
			Raise awareness with adolescents about the value of vocational and trade school professionals for society, e.g. via documentaries, media campaigns	Structure	Social media influencers; the Dutch Ministry of Education, Culture and Science; the Dutch Council for Secondary Education; School board of secondary schools
			Society should shift its emphasis away from the notion that e.g., college and university education equates to superiority compared to vocational and trade school education	Goal	Social media influencers; the Dutch Ministry of Education, Culture and Science; the Dutch Council for Secondary Education; School board of secondary schools
	**LP25:** Schools: maximizing cognitive learning (current Goal)	38	Adjustment of the core objectives and quality criteria that schools must adhere by	Structure	Ministry of Education, Culture and Science
			Schools should shift their focus from end-of-term exams and student enrolment numbers to prioritize the progress and well-being of adolescents	Goal	school board; Council for Secondary Education, Ministry of Education, Culture and Science
			Strengthen the belief that schools are there to facilitate learning, getting good grades and play a multifaceted role in promoting health among adolescents	Belief	the national Dutch Healthy School Program; Trimbos Institute
	**LP26:** Performance culture (i.e. you are responsible for your own life satisfaction and success) (current Belief)	39	The norm that 'success is not solely dependent on summative assessment' should be embedded in ministry policies	Structure	Council for Secondary Education, Ministry of Education, Culture and Science
			Ministry of Education, Culture & Science changes the norm that success is not solely dependent on summative assessment	Goal	Ministry of Education, Culture and Science
			Raise awareness among Ministry of Education and schools to counter the belief that one is solely responsible for his/her own mental wellbeing	Belief	Trimbos Institute; Council for Secondary Education; Ministry of Education, Culture and Science; Ministry of health, social welfare, and sport
		40	Implement public awareness (mass) media campaigns to increase awareness about the impact of performance pressure on adolescents' mental wellbeing	Structure	Trimbos Institute; the Dutch Ministry of Education, Culture and Science; local public health services; local municipalities; JOGG; the Dutch National Healthy School program; the Dutch Youth Institute; social media influencers
	**LP27:** Norm to not speak about mental wellbeing and seeking professional help (current Belief)	41	Normalize discussions about mental health problems among adolescents within mentor classes	Event	Schools
			Schools include health topics such as mental health within the school curriculum	Structure	School board
			Schools should foster an environment in which it is normal for adolescents to discuss mental health problems with friends, parents, and a psychologist, without any associated stigma. Educators have a guiding role in this	Goal	Schools
			Increase awareness among society that stigma on speaking about mental health is harmful for the mental health of adolescents	Belief	Trimbos Institute; the Dutch Youth Institute; (social) media influencers; the Dutch Ministry of Education, Culture and Science
**Digital environment**					
**Mechanism**	**Leverage Point**	**Action Number**	**Actions**	**ASM Level**	**Potential Implementers**
Postponing bed- and sleep timing	**LP28:** Providing adolescents with knowledge, awareness, positive attitude, self-efficacy and skills (e.g., to cope with FOMO and cultivating mechanisms) (Event)	42	Provide education regarding excessive screen use, the addictive techniques of social media platforms, and the effects on sleep	Event	Teachers
			Teach adolescents skills to cope with excessive screen use, e.g.,: • to set a push notifications to remind them to put your phone away, at for example 8:30 PM • (the advantages) to turn off (notifications on their) phone	Event	Teachers
			Schools include health topics such as screen use and social media within the school curriculum	Structure	School board
			Schools should focus on teaching (social) media literacy, particularly regarding the effects of social media persuasion	Goal	School board
			Raising awareness among schools about the importance and their influence on teaching (social) media literacy to strengthen the belief that (social) media literacy is important for the mental wellbeing of adolescents	Belief	Local public health services' healthy school advisors; the national Dutch Healthy School Program; JOGG; the Dutch Council for Secondary Education; the Dutch Youth Institute; Trimbos Institute; the Dutch Ministry of Education, Culture and Science; the Dutch Ministry of Health, Social welfare, and Sports
		43	Motivate adolescents to reduce their screen use	Event	(social media) influencers; peers; role models
			Role modelling techniques are implemented to get adolescents to reduce their screen time	Structure	(social media) influencers; peers; role models
		44	Teach children and adolescents from a young age, around 10 years old, to limit their screen time effectively	Event	(primary) schools
			Primary schools include topics such as (social) media and screen use within the school curriculum	Structure	School board
			Primary schools educate teenagers on topics such as (social) media and screen use	Goal	School board
			Raise awareness among primary schools about the importance and influence they have in teaching media literacy to teenagers. Emphasize the need to start as early as possible to strengthen the belief that inappropriate media and screen use can negatively affect both mental health and sleep quality	Belief	Local public health services' healthy school advisors; the national Dutch Healthy School Program; JOGG; the Dutch Council for Secondary Education; the Dutch Youth Institute; Trimbos Institute; the Dutch Ministry of Education, Culture and Science; the Dutch Ministry of Health, Social welfare, and Sports
	**LP29:** (social) media companies: the compulsive ‘structures’ or environments of media platforms by using personal digital data (Structure)	45	Adolescents are protected from the addictive properties that social media platforms employ to capture their continuous attention	Event	national Dutch government; social media companies
			Social media companies implement adjustments to their current techniques to keep users addicted to their platform(s), but rather employ techniques such as warnings, notifications, screen time tracking, autoplay features, and stop cues	Structure	Social media companies
			Get social media companies to quit using their digital addiction techniques	Goal	national Dutch government
			Raise awareness for the need to regulate the use of digital addiction techniques by social media platforms	Belief	Local public health services; the national Dutch Healthy School Program; the Dutch Council for Secondary Education; the Dutch Youth Institute; Trimbos Institute; Media education organisations (in Dutch: Bureau Jeugd & Media)
		46	Make adolescents aware of the compulsive 'structures' or environments	Event	Social media companies
			Develop and implement cautionary advertisement on social media platforms highlighting the disadvantages of screen use before bedtime	Structure	Social media companies
			Develop notifications and videos on social media platforms to raise awareness among adolescents about the importance of avoiding late-night screen use	Structure	Social media companies
		47	Parents use 'Family apps' that enable them to set time limits for their child's phone use	Event	Parents
			Parents receive information stressing the importance of limiting their child's bedtime screen time and practical tools to do so	Structure	Youth healthcare; Child and Family Services; School board; Dutch Youth Institute
			Aim to stimulate parents to reduce their children’s bedtime screen time to protect and improve their sleep health	Goal	Youth healthcare; Child and Family Services; School board; Dutch Youth Institute
			Strengthen the belief among parents that bedtime screen use disrupts adolescents' sleep habits, which in turn damages their physical and mental wellbeing as well as their school performance	Belief	Youth healthcare; Child and Family Services; School board; Dutch Youth Institute
	**LP30:** (social) media companies: (current Goal) • Maximize profits by increasing social media and screen use • Improve and maximize efficiency and convenience of daily life activities as the use of technology can simplify time-consuming tasks	N/A			
(peer) Norms sreen use & social media	**LP31:** School: Screen use at school for schoolwork contribute to task efficiency (but influences screentime/norms regarding screen use) (Structure)	48	The use of screens at schools takes place exclusively to support academic learning	Event	School board
			Schools implement policies that limit the use of smartphones and other screens for non-school purposes, such as anti-social media breaks or prohibition of phones at school	Structure	School board
			Schools receive support in order to implement screen reduction school policies	Structure	Local public health services' healthy school advisors; the national Dutch Healthy School Program; JOGG; the Dutch Council for Secondary Education; the Dutch Youth Institute
			Schools aim to allow screens in school only for academic purposes	Goal	School board
			Raising awareness among schools about the effects of limiting adolescents' screen use on establishing novel screen use norms and on students' social development	Belief	Local public health services' healthy school advisors; the national Dutch Healthy School Program; JOGG; the Dutch Council for Secondary Education; the Dutch Youth Institute; the Dutch Ministry of Education, Culture and Science; the Dutch Ministry of Health, Social welfare, and Sports
		11	See action #11 within ‘school environment’		
		16	See action #16 within ‘school environment’		
		17	See action #17 within ‘school environment’		
	**LP32:** Adolescents: Screen use (e.g., social media and gaming) are important communication ‘structures’ for teenagers. But also a way to explore their identity and express themselves (Structure)	N/A			
	**LP33:** Adolescents: conform with their peers and adhere to the social norm (current Goal)	N/A			
	**LP34:**Adolescent screen use norm, i.e., it is normalized to be online in the evening, it is expected that you are 24/7 available and that social media is an extension of our personal identity (i.e., many reactions, followers and likes is comparable to popularity and being liked) (current Belief)	49	Adolescents are subject to (mass media) information campaigns that focus on normalizing the "Joy Of Missing Out" (JOMO) and that denormalize the "Fear Of Missing Out" (FOMO)	Structure	Trimbos Insitute, The Dutch Ministry of Health, Social welfare, and Sports; local municipalities; the Dutch Youth Institute; JOGG; the Dutch National Healthy School program
		50	Encourage peer-to-peer discussions and conversations about the effects of screen usage on sleep health	Event	Teachers; the national Dutch Healthy School Program; Media education organisations
	**LP35:** Technological innovations are necessary to function in daily life (e.g. school work, entertainment, communication, payments, setting alarm clocks) (current Belief)	N/A			
**Personal system**					
**Mechanism**	**Leverage Point**	**Action Number**	**Actions**	**ASM Level**	**Potential Implementers**
Socio-cognitive factors (e.g., knowledge, awareness, attitude, self-efficacy and intention) as preconditions to stimulate healthy sleep behaviour	**LP36**: Providing adolescents with knowledge, awareness, positive attitude, self-efficacy and skills to improve their sleep health (Event)	51	Adolescents are taught about various aspects of sleep, including its importance and tips to improve their sleep hygiene practices and sleep health	Event	School board
			Schools implement sleep education in curriculum that highlight the benefits of sleep and risks of poor sleep. Preconditions: • E.g., through guest lectures by experts in sleep science • E.g., lessons need to be practical, interactive and preferably personal encouraging adolescents to reflect on and develop individualized sleep routines tailored to their needs, rather than enforcing a one-size-fits-all approach • E.g., incorporating video’s in sleep education to depict the consequences of sleep deprivation	Structure	School board
			Schools prioritize overall well-being alongside academic achievement	Goal	School board; the Dutch Council for Secondary Education Local public health services; the Dutch Ministry of Education, Culture and Science
			Raise awareness among schools about their significant influence on adolescent sleep health	Belief	Local public health services' healthy school advisors; the national Dutch Healthy School Program; JOGG; the Dutch Council for Secondary Education; the Dutch Youth Institute; the Dutch Ministry of Education, Culture and Science; the Dutch Ministry of Health, Social welfare, and Sports
		52	Adolescents are taught about various aspects of sleep, including its importance and tips to improve their sleep hygiene practices and sleep health	Event	Dutch Brain Foundation
			Developing and updating an informative website dedicated to educating adolescents about various aspects of sleep, including its importance and tips for improving sleep	Structure	Dutch Brain Foundation
			Create a reputable and trustworthy knowledge institute for the subject of ‘sleep’	Goal	the Dutch Brain Foundation, the Dutch Ministry of Health, Social welfare, and Sports
			Strengthen the belief among the Ministry of health, social welfare, and sport that adequate sleep health is important for society	Belief	Public Health Services, Dutch Brain Foundation
		53	Adolescents are taught about various aspects of sleep, including its importance and tips to improve their sleep hygiene practices and sleep health	Event	Schools
			Implement media campaigns, such as television commercials, to increase awareness about the importance of sleep	Structure	The Dutch Brain Foundation; the Dutch Ministry of Education, Culture and Science; local public health services; the Dutch Youth Institute; social media influencers
			Producing a documentary or series for streaming platforms like Netflix that delves into the detrimental effects of sleep deprivation on physical and mental health, exploring scientific research, personal stories, and expert insights to raise awareness about the importance of adequate sleep	Structure	The Dutch Brain Foundation; the Dutch Ministry of Education, Culture and Science; local public health services; the Dutch Youth Institute; social media influencers
		54	Reward adolescents when they perform good sleep-related behavior	Event	-
			Developing a sleep application that incentivizes users to adhere to consistent bedtime by awarding points for going to bed on time	Structure	Digital (mobile) software developers
		55	Primary school children are educated about sleep, stress and screen use	Event	Teachers
			Primary schools implement education about sleep, stress and screen use	Structure	School board
			Primary school should focus on topics as sleep, stress and screen use (i.e., teach adolescents as early as possible)	Goal	School board
			Increase awareness of primary schools about the importance and influence they have in teaching about sleep, stress and screen use. Emphasize the need to start as early as possible to strengthen the belief that inappropriate sleep, media and screen use and stress can negatively affect growing up	Belief	Local public health services' healthy school advisors; the national Dutch Healthy School Program; JOGG; the Dutch Council for Secondary Education; the Dutch Youth Institute; Trimbos Institute; the Dutch Ministry of Education, Culture and Science; the Dutch Ministry of Health, Social welfare, and Sports
Social norms about sleep health among adolescents	**LP37:** Influence specific social norms about sleep among adolescents, i.e. the norms (current Belief): • "Sleep is a waste of my time" • "Sleep is not important" • "Staying up late is cool" • "Peers also stay up late"	56	Exposing adolescents to information about the importance of sleep to make aware about the detrimental effects of inadequate sleep and thereby to change the norm of sleep is 'not cool'	Event	(social) media platforms
			Using influencer, social media and documentary structures to change the norm that sleep is 'not cool' (e.g., based on personal stories, scientific research and expert insights)	Structure	(social) media platforms
			Produce brief 3-min videos featuring influential figures such as musicians, actors, athletes, influencers, detectives, and military personnel discussing their sleep habits and emphasizing the importance of rest for optimal performance and well-being to change the current sleep norm	Structure	Role models for adolescents
			Utilize social media advertising to showcase both the benefits and drawbacks of inadequate sleep	Structure	The Dutch Brain Foundation
			Potential campaign developers should have the belief that it is important that teens value sleep health	Belief	N/A
		57	Parents are made aware that they are an important role model regarding sleep-related behavior, such as screen use and bedtiming. Thereby, performing desirable sleep-related behavior	Event	Youth healthcare; Child and Family Services; School board; Dutch Youth Institute
**Family- and home environment**					
**Mechanism**	**Leverage Point**	**Action Number**	**Actions**	**ASM Level**	**Potential Implementers**
Parenting practices	**LP38**: Aiding parents with setting, monitoring and enforce appropriate parenting practices (e.g. bedtime, sleep time and screen use) (Event) **LP39:** Providing parents with knowledge, positive attitude, awareness, self-efficacy and skills to improve sleep health of their children (Event)	58	Parents receive information, support and tools in context of child rearing from childhood to adolescence with a specific focus on supporting their child(ren)'s sleep health and screen use habits	Event	Youth healthcare; Child and Family Services; Dutch Youth Institute, the Dutch Brain Foundation; Local public health services
			Developing and updating an informative website dedicated to educating parents about various aspects of sleep, including tips for improving parenting sleep practices	Structure	Local public health services' healthy school advisors; the national Dutch Healthy School Program; JOGG; the Dutch Youth Institute, Child and Family services, youth health care
			Primary and secondary schools provide information, support and tools to students' parents in context of child rearing from childhood to adolescence with a specific focus on supporting their child(ren)'s sleep health and screen use habits	Structure	In context of both primary- and secondary schools: School board, Teachers, school healthcare professionals, local public health service's healthy school advisors
			Primary and secondary schools aim to support parents with child rearing issues, especially when related to sleep health and screen use habits	Goal	School board
			Dutch public health organizations aim to support parents with child rearing issues, especially when related to sleep health and screen use habits	Goal	Municipal departments of health, local public health services, the Dutch Youth Institute, JOGG
			Raise awareness with public health organizations and schools that parents need to be able to receive support in child rearing issues during the early adolescent years, especially with regard to contemporary issues as mobile phone use and sleep health	Belief	the national Dutch Healthy School Program; JOGG; the Dutch Youth Institute; the Dutch Ministry of Health, Social welfare, and Sports
		59	Parents receive information, support and tools in context of child rearing from childhood to adolescence with a specific focus on supporting their child(ren)'s sleep health and screen use habits	Event	Youth healthcare; Child and Family Services; Dutch Youth Institute, the Dutch Brain Foundation; Local public health services
			Dutch public health organizations provide information, support and tools to primary and secondary schools' students' parents in context of child rearing from childhood to adolescence with a specific focus on supporting their child(ren)'s sleep health and screen use habits	Structure	Municipal departments of health, local public health services, the Dutch Youth Institute, JOGG
			Having a national institute/organisation dedicated to research and policy in the area of sleep health	Structure	N/A
			Create a reputable and trustworthy knowledge institute for the subject of ‘sleep’	Goal	the Dutch Ministry of Health, Social welfare, and Sports
			Strengthen the belief among the Ministry of health, social welfare, and sport that providing accessible, reliable information on sleep health is important	Belief	the national Dutch Healthy School Program; JOGG; the Dutch Youth Institute; local public health services, the Dutch Brain Foundation
		60	Parents are exposed to social media campaigns designed to increase awareness about their children's sleep health	Event	-
			Create social media content to increase the awareness of parents about adolescent sleep health	Structure	Local and collaborative public health services; the national Dutch Healthy School Program; the Dutch Youth Institute; the national Dutch government
			Raise awareness with public health organizations that parents need to be informed and supported on the issue of adolescent sleep health	Belief	the national Dutch Healthy School Program; JOGG; the Dutch Youth Institute; the Dutch Ministry of Health, Social welfare, and Sports
	**LP40:** Aiding parents to be a good role model (e.g. parental evening screen use, bedtime) (Event)	61	Parents are subject to a campaign to increase parental awareness on how their sleep-related behavior (e.g., screen use and bedtiming) set the norm for their children and influence their sleep-related behavior	Event	-
			Developing a campaign to increase parental awareness on how their sleep-related behavior (e.g., screen use and bedtiming) set the norm for their children and influence their sleep-related behavior	Structure	Local and collaborative public health services; the national Dutch Healthy School Program; the Dutch Youth Institute; the national Dutch government, the Dutch Brain Foundation
		62	Parents should serve as positive role models for adolescents by encouraging open communication about mental well-being	Event	Parents
	**LP41:** Creating accessible ways for parents to obtain information, guidance and exchanging experiences with other parents (Structure)	59	See action #59 within ‘family- and home environment’		
		63	Parents receive information, support and tools in context of child rearing from childhood to adolescence with a specific focus on supporting their child(ren)'s sleep health and screen use habits	Event	Youth healthcare; Child and Family Services; Dutch Youth Institute, the Dutch Brain Foundation; Local public health services
			Provide both online and physical information structures to enable anonymity (e.g., websites, podcasts, workshops, parenting evenings, e-learnings)	Structure	
			Develop occasions where parents can receive information, tips on parenting and engage with other parents (e.g., parenting festivals/evening information sessions at school)	Structure	Schools; Local and collaborative public health services
			Schools organize host themed parent evenings to strengthen parental support with a focus on addressing concerns such as performance pressures and mental health issues related to their children and providing tips & tricks	Structure	Schools
	**LP42**: Parental norm around sleep and screen parenting (current Belief)	64	Parents receive more information about the standards regarding sleep parenting practices	Event	-
			Schools provide information about puberty and health preferably in primary school or the first year of secondary school	Structure	Schools
		61	See action #61 within ‘family- and home environment’		
	**LP43:** Seeking parental help is perceived as failing as a parent (current belief)	63	See action #63 within ‘family- and home environment’		
Household factors	**LP44:** Adolescents at this age range should take their own responsibility, or adolescents at this age range can’t take all responsibilities and still need a guiding role from their parents (current Belief)	20	See action #20 within ‘school environment’		
	**LP45:** Improving municipal access to financial resources for low socio-economic status families to enable them to create a healthy sleep environment (Structure)	N/A			
	**LP46:** Busy day to day lives (less priority for parenting practices and less insight into adolescent behavior) (Structure)	63	See action #63 within ‘family- and home environment’		
Other	**LP47**: Parents: their teenagers should grow up happily and in good physical and mental state (current Goal)	N/A			
	**LP48:** Parents: do good as a parent and facilitate the best possible future for their child (current Goal)	N/A			
	**LP49:** Parents: have a good relationship with their child (current Goal)	65	Create a digital application designed to showcase the current trending apps among adolescents. This tool serves as a platform for facilitating constructive dialogues between parents and their children regarding social media engagement	Event	-
		66	Provide parents with more information on improving on how to better connect with their child, facilitated through school initiatives, coaching, educational modules and national programs	Event	Youth healthcare; Child and Family Services; Dutch Youth Institute, the Dutch Brain Foundation; Local public health services
	**LP50:** Parents: having the highest educational level possible for their child (current Goal)	N/A			
	**LP51:** Parental belief that higher educational qualifications of their child enables financial independence and betters their chances in life (current Goal)	N/A			

All co-creation sessions followed the same structure (Additional file I). *Prior* to these sessions, participants received a ‘sensitizer’ – namely, a short (online) questionnaire designed to stimulate participants to think about the topic before joining the co-creation session (i.e., “What should schools do regarding the topic of stress and sleep?”). This served for participants to think about their own experiences and formulate their own opinions before engaging in group discussions. Then, *during* each session, individuals first met in homogenous actor groups (i.e., adolescents grouped together, parents in another, and professionals also in their respective peer groups) to brainstorm and identify numerous actions aimed at targeting the previous identified leverage points for that specific subsystem (e.g., come up with solutions to change evening school notifications). Participants were actively stimulated to think of potential actions in context of the different system levels of the ASM model facilitated by prompt actions and questions. For example, based on the example shown in Fig. 2: “What can you do personally? What can teachers/school leaders/the local government/the Ministry of Education do? How can we change the school structure? How can we change the current norm/attitude about successful citizenship?”. Thereafter, participants prioritized what they felt to be the most impactful, promising, fitting actions per leverage point. Subsequently, three to four multi-actor groups were formed. Participants collectively chose one idea to further develop. Thereafter participants shared their ideas with others and received feedback and suggestions for improvement from other groups.

Throughout all the data collection efforts, we iteratively identified and learned from the sessions’ strengths and weaknesses to improve on their design [25]. This enhanced continual learning throughout the process. This, for example, refined session details with regards to the ideal number of participants for effective discussion or how to create the optimal atmosphere to empower all participants to engage.

### Co-creation phase 2: Identifying potential implementers

Between June and October 2022, three co-creation sessions were held using the World Café Methodology, each lasting 2 h. Their aim was twofold:

1. identify and clarify additional actions/ideas targeting one or more leverage points;
2. identify who/what organization can structurally implement the conceived actions

Using the World Café methodology, participants gathered in a comfortable and informal setting where they received information about the process. After being divided into small groups at different tables, discussions took place in timed rounds (e.g., 20–30 min), focusing on a specific question or topic. Every table was focused on one of the five sleep subsystems. Printed posters were used to illustrating the previous identified system dynamics per subsystem [6] supplemented with the actions as identified in phase 1. Participants reflected on additional/existing ideas as well as potential implementers. Each round began with a context-specific *question* designed to steer the conversation (e.g., “please look at this part of the poster, do you miss an action to prevent late night school notifications?”). After each round, members moved to a new table while the ‘table host’- either a facilitator or participant- stayed behind to welcome the next groups and maintain the conversation’s flow. Finally, everyone reconvened to share and synthesize the most important insights and ideas emerged from the discussions (i.e., in this study the facilitator presented the results to the rest of the group whereafter participants could respond).

#### Data analysis

First, all actions of the phase 1 co-creation sessions were structured according to the leverage points they aimed to target. Thereafter, actions were thematically analyzed and similar or duplicate ideas were removed. All actions proposed were given equal weight, with no distinction between the actor groups. Actions that were not considered feasible (e.g., paying teenagers to go to bed earlier) were not included in the action plan. Third, the actions/ideas were written out in full and integrated into the previously developed Causal Loop Diagram using Kumu software (Kumu relationship Mapping Software 2024). With this, the actions were linked to the specific leverage point they aimed to address. Then, after each World Café session, actions/ideas and potential implementers were thematically analyzed and additional actions were added to the Causal Loop Diagram using Kumu. Additionally, the session notes were analyzed, from which additional actions were derived. All actions were categorized according to the Action Scales Model levels (i.e., event, structure, goal, belief) to gain insight into which levels of the system the actions were co-created for. Sets of actions across different ASM levels were numbered and integrated within the CLD at the points where they influence a factor or disrupt a mechanism. All phases of the analysis were conducted and discussed independently by at least two researchers.

## Results

The results of the co-creation sessions, both phase 1 and phase 2, are visually depicted in Table 1. Specifically, a resulting whole systems action plan to promote sleep health of Dutch adolescents is presented whereby the reader can find an overview of all mechanisms and leverage points per subsystem, newly identified actions; their ASM classification; and (potential) implementers. This plan is visually depicted in Fig. 1 whereby the original CLD developed by Heemskerk et al. [6] is supplemented with actions identified during the co-creation sessions. In total 66 sets of actions emerged targeting the leverage points within the following subsystems: school environment [yellow] *N* = 24; mental wellbeing [mint green] *N* = 17; digital environment [orange] *N* = 9; family & home environment [purple] *N* = 9.; personal system [red] *N* = 7).

**Fig. 3:**
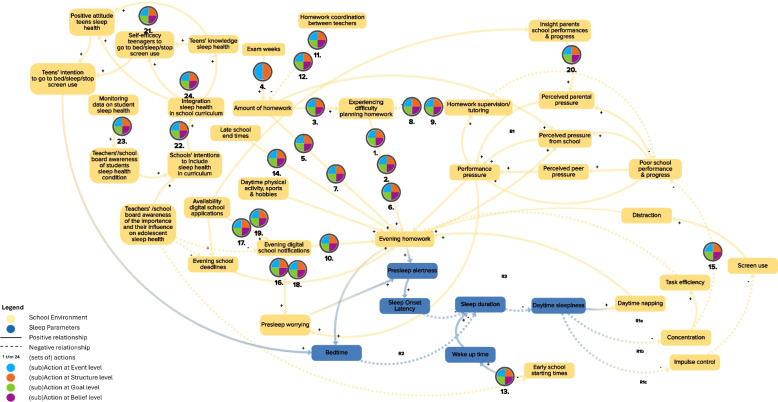
Causal loop diagram of the school environment subsystem of adolescent sleep health, including all potential whole system action plan actions (adapted figure from Heemskerk et al. [6]

These actions are organized into coherent sets each jointly targeting a specific identified leverage point. Typically, such a set consists of actions across different ASM levels. Actions are numbered in the Figure and Table to ensure correspondence, and to further aid interpretation, are placed within the CLD at the points where they influence a factor or disrupt a mechanism. The sets of actions are visualized with colored dots representing the sub-actions and their ASM classification. For example, action #1 represents four sub-actions across the ASM level (i.e., event = blue, structure = orange, goal = green, belief = purple) (Fig. 1 & Table 1). Figure 3 visualizes the relationships, feedback loops and leverage points within the subsystem ‘school environment’. Figures of the other subsystems can be found in Additional file 2–5.

### Whole systems action plan

While Table 1 and all figures comprehensively represent all aspects of the co-designed whole systems action plan for Dutch adolescent sleep health, we offer an example of how to interpret the results here as a guidance to the reader. Specifically, to start, it is important to select the leverage point of interest. For example, Heemskerk et al. [6] previously found that the leverage point ‘*no digital communication from schools in the evening*’ (subsystem: school environment) has considerable impact on adolescent sleep health, since they found that schools in the Netherlands often send messages to adolescents about grades, schedule changes and other school-related information late in the evening. A total of six sets of actions were identified to influence this leverage point, specifying for each sub-action which ASM level it targeted and what potential actor(s) would be best suited to implement each action in practice. For example, one broad set of actions focused on policies at the schools and was ultimately reflected across several sub-actions reflecting different ASM levels as denoted in Table 2.

**Table 2:** Sample Actions for Leverage Point “no digital communication from schools in the evening

Sample Actions for Leverage Point *“no digital communication from schools in the evening”* focusing on school policy	Level	Potential implementer
‘Students do not receive any digital notifications from school and they cannot access the online grading and test scores system between 8:00 PM and 8:00 AM’	Event	School board
'Schools implement policies that prevent sending students notifications as well as to prevent them from accessing the online test scores and grading system between 8:00 PM and 8:00 AM’	Structure	School board
'Schools are provided with tools and guidance such as policy advice templates and good practices examples on how to implement restrictions related to digital communication between teachers and adolescents in the evening’	Structure	Local public health services' healthy school advisors; the national Dutch Healthy School Program; JOGG; the Dutch Youth Institute
‘Schools aim to protect students' mental wellbeing and prevent stress’	Goal	School board
‘Schools are made aware that evening notifications (e.g. communicating test results) causes students stress that in turn negatively affects their sleep health’	Belief	Local public health services' healthy school advisors; the national Dutch Healthy School Program; JOGG; the Dutch Council for Secondary Education; the Dutch Youth Institute
‘Raise awareness among schools that their students have the right to leisure time after school hours to keep a healthy school-private life balance and protect their mental wellbeing’	Belief	Local public health services' healthy school advisors; the national Dutch Healthy School Program, the Dutch Council for Secondary Education; the Dutch Youth Institute; the Dutch Ministry of Education, Culture and Science; the Dutch Ministry of Health, Social welfare, and Sports

To ensure sustainable change, coherence between the different system levels is required. However, this does not always mean that intervention efforts at all system levels are always needed. In this example, as shown in Table 2, simultaneous actions at four levels (i.e., event, structure, goal, belief) are needed in order to reduce the likelihood adolescents receiving late-night school notifications recurring in the future. In addition, regarding this specific leverage point, six sets of actions were identified. That said, ideally, the recommendation in this case would be to not select one set of actions, but rather to aim for the leverage point from as many angles as possible – thus leading to the greatest likelihood of change.

## Discussion

Using a co-creation methodology with adolescents, parents, and professionals, together with the Action Scales Model, this study presents what is believed to be the first whole systems action plan for improving the sleep health of Dutch adolescents. This resulted in a coherent total of 66 designed actions and identified potential implementers within the Dutch context across five sleep health subsystems: school environment (*N* = 24), mental wellbeing (*N* = 17), digital environment (*N* = 9), family & home environment (*N* = 9), personal system (*N* = 7). Furthermore, we visualized how these actions are distributed across the four Action Scales Model levels.

### A whole systems action plan to promote adolescent sleep health

Several recent studies have advocated for a more holistic approach by targeting multiple socio-ecological levels across diverse contexts (e.g., family, schools, digital) in order to promote adolescent sleep health [5–7, 26]. However, an umbrella review examining the scope of current sleep health interventions showed that, except for some studies targeting later school start times, most existing interventions target adolescents themselves as main actors to realize behavioral changes (i.e., sleep education, relaxation techniques, psychotherapy) [7] – highlighting the need to develop a more comprehensive way to effectively and sustainably impact adolescent sleep health, e.g., via a whole systems action plan.

As demonstrated in our current study, impacting adolescent sleep health requires a holistic approach and a coherent set of actions involving collaboration among multiple actors from different sectors on local and national level in line with the WHO’s Health *in* All Policies (HiAP) framework. This means focusing on promoting health and health equity through policies that extend beyond the public health and healthcare sector [27] in order to create more impactful, structural systems changes. Using such a HiAP approach, the ‘Educational’ policy domain, for example, can (potentially) contribute to e.g., delaying school starting times, supporting schools in creating healthy school environments and integrating sleep health into the school curriculum. Additionally, taking a ‘Health *for* All Policies’ point of view, improving sleep health can be used as a tool instead of a means and goal in itself to benefit other “sectors” such as education [28]. This way, improving sleep health can be framed in the political debate and in policy design as a tool to stimulate e.g. mental wellbeing, school engagement, school performance, school-related burnout, absenteeism, and bullying [29], all core targets for the ‘Educational’ and ‘Youth and Welfare’ domains. Emphasizing the co-benefits from improving health outcomes, such as sleep health, can serve as a stimulant for intersectoral collaboration.

### Using the action scales model to facilitate systems change

To develop the whole systems action plan, we applied the Action Scales Model. We considered the ASM as a bridge between systems science and participatory approaches, facilitating both the process of identifying coherent sets of actions across different system levels and making systems science principles and methods achievable during our co-creation sessions. For example, we used the model prospectively by framing guiding questions aimed at prompting participants to consider actions across multiple system levels. This seemed especially useful considering that such systems thinking is quite unfamiliar for adolescents, parents and, generally, most professionals. However, we recommend developing tools to enhance the accessibility of the Action Scales Model (ASM) for a broader audience. This will enable a wider range of target groups, such as practitioners and policymakers, to utilize the model independently, without needing assistance from researchers and professionals familiar with it. Like many models, the ASM can be seen as somewhat arbitrary, depending on its application and interpretation (e.g., the subjectivity involved in level selection). For example, in this study, it became clear that actors involved identified policy as a primary method for altering structures within their context. Additionally, increasing awareness emerged as a crucial first step in transforming beliefs, norms and attitudes. By developing tools and encouraging more studies to apply and operationalize the ASM, we can make the model more accessible. This, in turn, empowers stakeholders to effectively implement the model in their fields, make informed decisions, and ultimately drive more impactful, evidence-based outcomes.

Using the ASM, it stood out that a larger number of actions were identified at the ‘event’ and ‘structure’ levels than were at the ‘goal’ and ‘belief’ levels. This is unsurprising as these are almost by definition the more visible and tangible system elements in comparison to the goals and beliefs that shape the deeper levels of the system. Moreover, developing a whole systems action plan is an extensive, iterative process. Initially, to properly understand a complex health behavior such as sleep health, the lived experiences of the target population and those closely involved with them are usually aimed to be understood. These lived experiences often involve tangible, visible elements or events and structures, as is the case with the often-used metaphor of ‘the tip of the iceberg’. Actions that address underlying goals and beliefs can then be determined by asking why these events and structures occur. However, the actors who have an influence on such deeper system goals and beliefs are often different actors than those who were initially brought in to uncover impactful events and structures. Therefore, they are often not yet involved in the earlier iterations of a whole systems action plan such as the one designed within our current study (e.g., legislators, policymakers, school boards). This explains why some underlying beliefs and system goals have not yet been concretely targeted in our whole system action plan and/or why some actions on these levels do not have concrete implementation actors connected to them yet. The design of a whole systems action plan should always be considered as iterative and long-term oriented with the intention of including more and more actors at the deeper levels.

Lastly, it is important to emphasize that the coherence across all potential actions is of relevance instead of quantifying the amount of actions or ensuring that all ASM levels are always targeted regardless of context. What matters most is that all relevant levers on all system levels are aligned to positively impact adolescent sleep health. This was illustrated in the aforementioned example of digital school communication in the late evening. It showed that not every set of actions required changes across all four ASM levels; sometimes, simply adjusting a particular structure was enough to initiate the necessary changes; however, in other cases, actions across multiple levels were required, depending on the specific situation. The ASM emphasizes the importance of alignment across all levels, ensuring that each level supports the system in moving cohesively in the same and desired direction.

### Looking ahead

To our knowledge, this study is the first to create a whole systems action plan, informed by a previous comprehensive understanding of the system with multiple actors using the ASM, targeting adolescent sleep health. By doing so, it expands both the body of literature concerning the operationalization of whole systems approaches in addressing public health challenges [9] as well as on sleep health promotion in public health.

Our co-creative systems approach poses a considerable methodological strength, as it contributes to a comprehensive whole systems action plan that both addresses the root causes of poor adolescent sleep health and is closely aligned with the lived experiences of the target populations and the most relevant actor groups, ensuring that the proposed actions are both practical attainable and implementable. That was partly attributable to the multi-stakeholder approach of the co-creation sessions which facilitated consensus, resulting in actions that were not solely from either the adolescents or the adult actors, but rather collaborative efforts. By including both perspectives that are closely aligned with the adolescent and those focused on the implementation structures of actions and policies we feel that we have gained a fairly complete action plan. However, as mentioned above, developing an action plan remains an iterative process. We noticed that to co-create actions at deeper system levels (e.g., goals, beliefs), such as policies, involving additional actors (e.g., legislators, policymakers, school boards) is required. To further develop and refine effective implementation strategies for these type of actions, it would be desirable to include these key actors in a follow-up step.

Similar to our previous study mapping the system dynamics shaping adolescent sleep health within the Dutch context [6], we expect that the actions targeting the leverage points may be generalizable to other contexts but the content of the actions and coherence between the sub-actions and their ASM classification may differ dependent on the context (e.g., no action at belief level is needed in this context). With this in mind, for researchers interested in developing interventions promoting sleep health in communities other than the Dutch adolescent context, we recommend replicating our steps to understand the sleep health system of interest and develop a whole systems action plan addressing mechanisms within this system. In situations where resources and time are limited and replication not possible, we suggest that careful consideration be given to the context and that stakeholders with knowledge about the situation help select those leverage points that seem most context-agnostic and/or most consistent with the context at hand.

While we hope to see scholars replicate and extend this work in their own communities, for us, the next step is implementation of this plan. The action plan entails different actions across different settings. Its added value lies in viewing it as one whole to be executed coherently rather than addressing its isolated parts per setting. However, the action plan cannot be implemented all at once. It will be important to prioritize actions that are coherent, feasible and have significant leverage to transform the system into the desired situation. This involves examining the current ‘window of opportunity’, such as heightened public awareness, political support or momentum, available funding, or pressing needs that could guide and facilitate decisions about which leverage points and actions to focus on. However, there is not always a window of opportunity available. In such cases, it becomes essential to actively create one by developing and implementing strategic actions that establish the ‘problem stream’ and catalyze agenda setting, policy formulation and policy action. This process may involve raising awareness about pressing issues, shifting public opinion, and engaging stakeholders to highlight the urgency and significance of these challenges. After prioritizing, the action plan should be translated into an action program including a cohesive package with systematically developed interventions and implementation strategies. Preferably the development of these actions and implementation strategies should be based on theory and scientific evidence to increase the likelihood to achieve the desired change by using structured intervention development and implementation approach such as Intervention Mapping [30] or the Behavior Change Wheel [31].This also means considering the timeframe as a parameter for decision, especially since actions on event and structure level may be more achievable on the short term (e.g., providing adolescents with knowledge, awareness, positive attitude, self-efficacy and skills to improve their sleep health) while actions at goal and belief level may take up for several years (e.g., changing beliefs regarding performance culture). To do this, intersectoral collaboration will be essential [8, 9]. Even more, for sustainable implementation, it will be crucial to have a ‘problem owner’ or joint ownership to maintain momentum and drive the implementation of actions needed to improve adolescent sleep health [12]. An example of this can be observed in Whole Systems Approaches (WSAs) to obesity, such as the ‘Amsterdam Healthy Weight Approach’ [8] and the ‘Whole Systems Approach to Obesity [12]. Both are long-term, local authority-led, multi-sectoral WSA’s aimed at reducing overweight and obesity. These WSAs seek to strengthen and align with the existing strategies and infrastructures of local authorities. Adding to implementation, it will be important to evaluate how the actions contribute to systems change and to facilitate program adjustments as necessary in response to these systems changes in the future [8].

## Conclusion

This study provides details on the development and content of a whole systems action plan promoting Dutch adolescent sleep health providing insights into *which* leverage points to target, *how* to do this, and *who/what* is needed to realize that. A coherent total of 66 actions and potential implementers were identified within the Dutch context across five sleep health subsystems: the school environment, mental wellbeing, digital environment, family & home environment, and the personal system. The combination of a systems approach, using the Actions Scales Model, and a co-creation approach was found to be mutually reinforcing and helpful in developing a coherent, feasible and comprehensive whole systems action plan. This study contributed to the expanding body of literature concerning the operationalization of whole systems approaches tackling public health challenges and can be used as a guidance for strategic planning and implementation of actions that promote systemic change within the adolescent sleep health system. With this, it is important to ensure coherence between actions being developed and implemented to increase the potential for lasting systems change.

## Supplementary Information

Additional file 1. Description of co-creation session structure. 

Additional file 2. Causal loop diagram of the mental wellbeing subsystem of adolescent sleep health, including all potential whole system action plan actions (adapted figure from Heemskerk et al. [6].

Additional file 3. Causal loop diagram of the digital environment subsystem of adolescent sleep health, including all potential whole system action plan actions (adapted figure from Heemskerk et al. [6].

Additional file 4. Causal loop diagram of the family & home environment subsystem of adolescent sleep health, including all potential whole system action plan actions (adapted figure from Heemskerk et al. [6].

Additional file 5. Causal loop diagram of the personal subsystem of adolescent sleep health, including all potential whole system action plan actions (adapted figure from Heemskerk et al. [ 6].

## Data Availability

The datasets used and/or analysed during the current study are available from the corresponding author on reasonable request.

## Abbreviations

ASM: Action Scales Model
CLD: Causal Loop Diagram
WSA: Whole Systems Approach

## Footnotes

1 The Dutch education system: Pupils entering secondary education in the Netherlands (usually around 12 years of age) are streamed according to aptitude into one of four forms of schooling: practical education (PrO), pre-vocational education (*vmbo*), senior general secondary education (*havo*), and pre-university education (*vwo*).

## Glossary

**Causal mechanism**: Refers to the process or set of processes explaining why certain outcomes (such as inadequate sleep health) occur as a result of specific inputs or conditions [32].
**Causal Loop Diagram (CLD)**: Visual representation of a system and its dynamics visualizing factors, their interrelationship and feedback loops [33].
**Action Scales Model (ASM)**: Framework for understanding and addressing complex problems by distinguishing multiple levels (i.e., events, structures, goals and beliefs) to analyze a system and to intervene within a system to initiate systems change [13].
**Leverage points**: Modifiable points within a system. When these points are altered, this could lead to changes in the functioning, and thus the (health)outcome, of the system [34].
**Whole Systems Approach (WSA)**: An approach to address complex problems by considering all interconnected factors and relationships within an entire system to develop holistic and sustainable solutions [9].
**Whole Systems Actions Plan**: A plan/strategy that addresses complex problems by integrating and coordinating actions coherently across different system levels, subsystems and settings to achieve systemic, sustainable systems change [12].
