# Supplementary material for: Developing a whole systems action plan promoting Dutch adolescents’ sleep health
Source: Int J Behav Nutr Phys Act. 2025 Mar 17;22:33. doi: 10.1186/s12966-025-01711-0 (PMC11917006; doi:10.1186/s12966-025-01711-0)
Supplement: Supplementary file 1 — Additional file 1. Description of co-creation session structure. [file 12966_2025_1711_MOESM1_ESM.doc]

**Additional file 1.** Description of co-creation session structure

| **Structure co-creation sessions** | **Aim** | **Group composition** |
| --- | --- | --- |
| *Prior: sensitizer* | Short online questionnaire prompting participants to contemplate the topic independently, fostering personal opinions before starting a group discussion, and reducing social desirability bias. Sensitizer questions were formulated based on the topics of the leverage points discussed in the session, e.g., “I believe that schools and teachers should refrain from entering grades into the school app during the evening”. | Individually |
| 1. Introduction and acquaintance | - Introducing the facilitators. - Explaining the purpose of the co-creation session. - Introducing the participants via a warming up exercise (e.g., “arrange yourselves in alphabetical order according to your first names” or “organize yourselves according to the duration of your sleep last night”). This ‘icebreaker exercise’ was aimed to create a comfortable atmosphere for participants. | Collective |
| 2. Co-creation phase 1: identifying actions | - Introducing the leverage points of the co-creation. - Participants should brainstorm numerous actions to transition from the current situation to the desired state for each leverage point. They wrote down their ideas on canvas pages with the title: “ideas and solutions [topic – e.g., evening school notifications], write your ideas on post its”. To encourage participants to think about actions at different levels, facilitators asked prompt questions. For example, within the school domain: “What can you do personally? What can teachers do? What can school leaders do? What can the local government do? What can the Ministry of Education do?”. - Participants prioritize the best action per leverage point based on feasibility and impact. | Homogenous subgroups |
| 3. Co-creation phase 2: co-create actions | - Participants were mixed to form heterogeneous subgroups with at least 2 adolescents per subgroup. - Each subgroup was assigned one leverage point they feel most affiliated with. They collectively chose one prioritized action of phase 1. - Participants translated the action into a concrete plan of action considering the following questions:   - “What is the idea? Write your idea in a maximum of 3 sentences.”   - “Where is the idea to be implemented/executed?”   - “Who is needed to conduct the action(s) and what support is needed to achieve this? Think about money, people, materials etc.”   - “How do you involve the desired people to implement this idea?”   - “What are the problems you may run into? And how do you plan to solve them?”   - “What does the idea look like when translated into such concrete action(s)?”   - “Do you have any additional idea, questions or thoughts related to the idea?” | Heterogeneous subgroups |
| 4. Co-creation phase 3: validating actions | - Participants were asked to visit the tables of the other groups. Facilitators remained and elaborated on the ideas and action to the ‘new’ participants who just joined their table. - Participants were asked to provide tips and tops on the ideas and actions of the table they newly joined, and thereafter to refine them. The facilitators asked questions such as: “What rating from 1 to 10 would you give this idea? Why do you give it that score? What is needed to improve this idea to a 9 or 10? Are you missing any important information? Do you think this idea will work and really influence the mechanism/factor it is meant to influence? Would you, your friends, parents, teachers, or school use this idea? What could be improved and/or done differently?”. | Heterogeneous subgroups |
| 5. Closing | - A plenary discussion is held to examine whether the same findings emerged from different actor perspectives. This allowed them to complement each other one final time and/or refine their ideas to reach a consensus. - The facilitators provide an explanation of the subsequent actions in developing a WSA improving adolescent sleep health. - The facilitators thank the participants for all their input and asked whether the participants had any final additions to all that was produced and discussed, whereafter they asked what they thought of the session and whether there were any processes might be improved when doing this again in the future. | Collective |
| *After session* | - Facilitators documented their notes, and discussed and documented notable aspects of the session, including topics as:   - Setting of the session;   - Description of the participants   - Interaction between the interactions, atmosphere within the group   - Notable aspects of the session   - Key outcomes of the session   - Surprising elements of the session   - Insights to take into the next session | Research team |
