# Supplementary material for: Developing a whole systems action plan promoting Dutch adolescents’ sleep health
Source: Int J Behav Nutr Phys Act. 2025 Mar 17;22:33. doi: 10.1186/s12966-025-01711-0 (PMC11917006; doi:10.1186/s12966-025-01711-0)
Supplement: Supplementary file 2 — Additional file 2. Causal loop diagram of the mental wellbeing subsystem of adolescent sleep health, including all potential whole system action plan actions (adapted figure from Heemskerk et al. [6]. [file 12966_2025_1711_MOESM2_ESM.pdf]

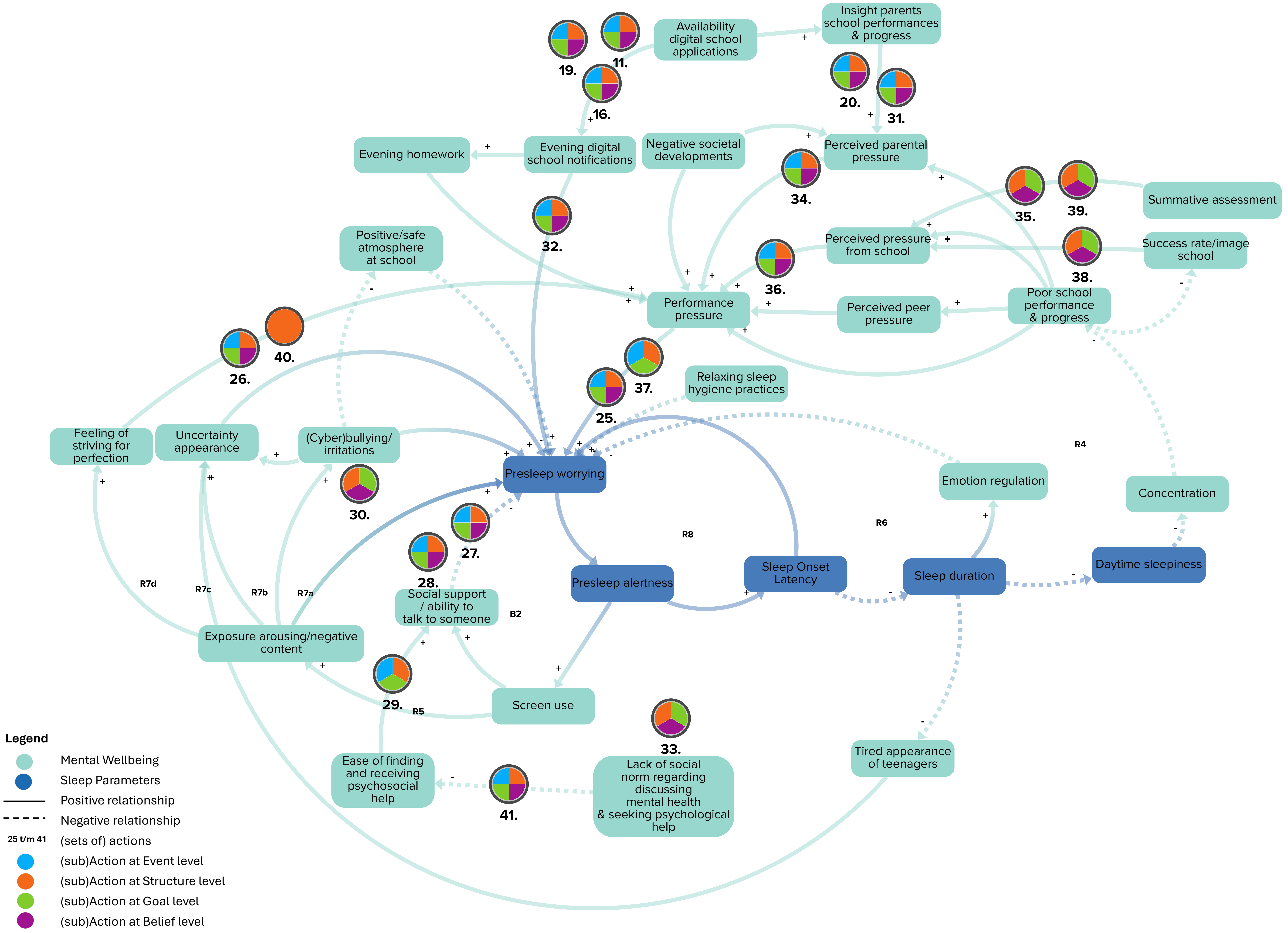

Availability digital school applications
Insight parents school performances & progress
Evening homework
Evening digital school notifications
Negative societal developments
Perceived parental pressure
Summative assessment
Positive/safe atmosphere at school
Perceived pressure from school
Success rate/image school
Performance pressure
Perceived peer pressure
Poor school performance & progress
Relaxing sleep hygiene practices
Feeling of striving for perfection
Uncertainty appearance
(Cyber)bullying/ irritations
Presleep worrying
Emotion regulation
Concentration
R4
R6
R8
Daytime sleepiness
Sleep duration
Sleep Onset Latency
Presleep alertness
Social support / ability to talk to someone
B2
R7d
R7c
R7b
R7a
Exposure arousing/negative content
Screen use
R5
Ease of finding and receiving psychosocial help
Lack of social norm regarding discussing mental health & seeking psychological help
Tired appearance of teenagers
19.
11.
16.
20.
31.
34.
35.
39.
32.
36.
38.
40.
26.
37.
25.
30.
27.
28.
29.
33.
41.
Legend
Mental Wellbeing
Sleep Parameters
Positive relationship
Negative relationship
25 t/m 41
(sets of) actions
(sub)Action at Event level
(sub)Action at Structure level
(sub)Action at Goal level
(sub)Action at Belief level
