# Supplementary material for: Developing a whole systems action plan promoting Dutch adolescents’ sleep health
Source: Int J Behav Nutr Phys Act. 2025 Mar 17;22:33. doi: 10.1186/s12966-025-01711-0 (PMC11917006; doi:10.1186/s12966-025-01711-0)
Supplement: Supplementary file 3 — Additional file 3. Causal loop diagram of the digital environment subsystem of adolescent sleep health, including all potential whole system action plan actions (adapted figure from Heemskerk et al. [6]. [file 12966_2025_1711_MOESM3_ESM.pdf]

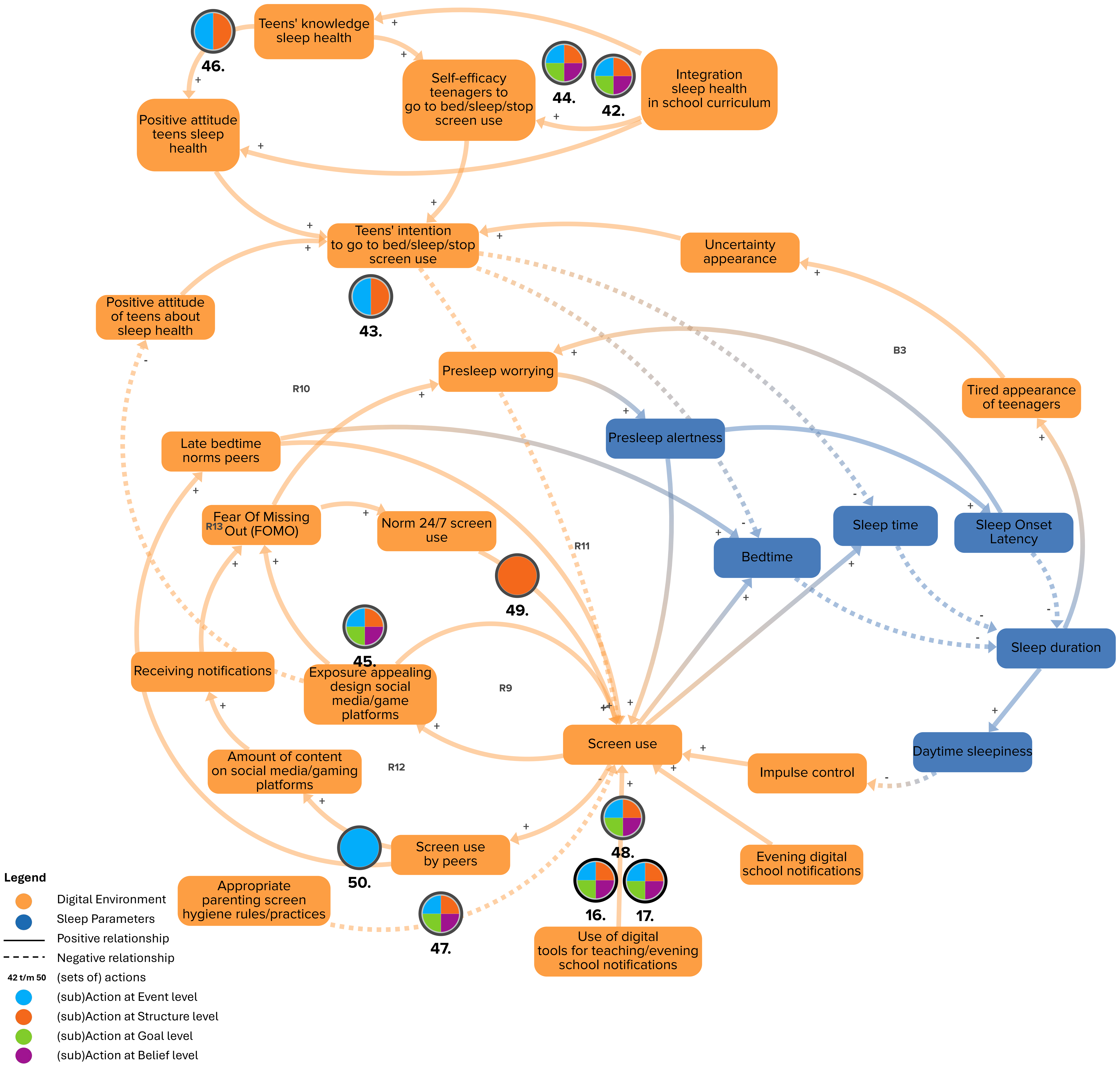
Teens' knowledge sleep health
Integration sleep health in school curriculum
Self-efficacy teenagers to go to bed/sleep/stop screen use
Positive attitude teens sleep health
46.
44.
42.
Teens' intention to go to bed/sleep/stop screen use
Uncertainty appearance
43.
Positive attitude of teens about sleep health
B3
R10
Presleep worrying
Tired appearance of teenagers
Presleep alertness
Late bedtime norms peers
Fear Of Missing Out (FOMO)
R13
Norm 24/7 screen use
R11
Bedtime
Sleep time
Sleep Onset Latency
49.
Sleep duration
45.
Receiving notifications
Exposure appealing design social media/game platforms
R9
Screen use
Daytime sleepiness
Impulse control
Amount of content on social media/gaming platforms
R12
48.
Screen use by peers
50.
Evening digital school notifications
16.
17.
Appropriate parenting screen hygiene rules/practices
47.
Use of digital tools for teaching/evening school notifications
Legend
Digital Environment
Sleep Parameters
Positive relationship
Negative relationship
42 t/m 50 (sets of) actions
(sub)Action at Event level
(sub)Action at Structure level
(sub)Action at Goal level
(sub)Action at Belief level
