# Supplementary material for: Developing a whole systems action plan promoting Dutch adolescents’ sleep health
Source: Int J Behav Nutr Phys Act. 2025 Mar 17;22:33. doi: 10.1186/s12966-025-01711-0 (PMC11917006; doi:10.1186/s12966-025-01711-0)
Supplement: Supplementary file 4 — Additional file 4. Causal loop diagram of the family & home environment subsystem of adolescent sleep health, including all potential whole system action plan actions (adapted figure from Heemskerk et al. [6]. [file 12966_2025_1711_MOESM4_ESM.pdf]

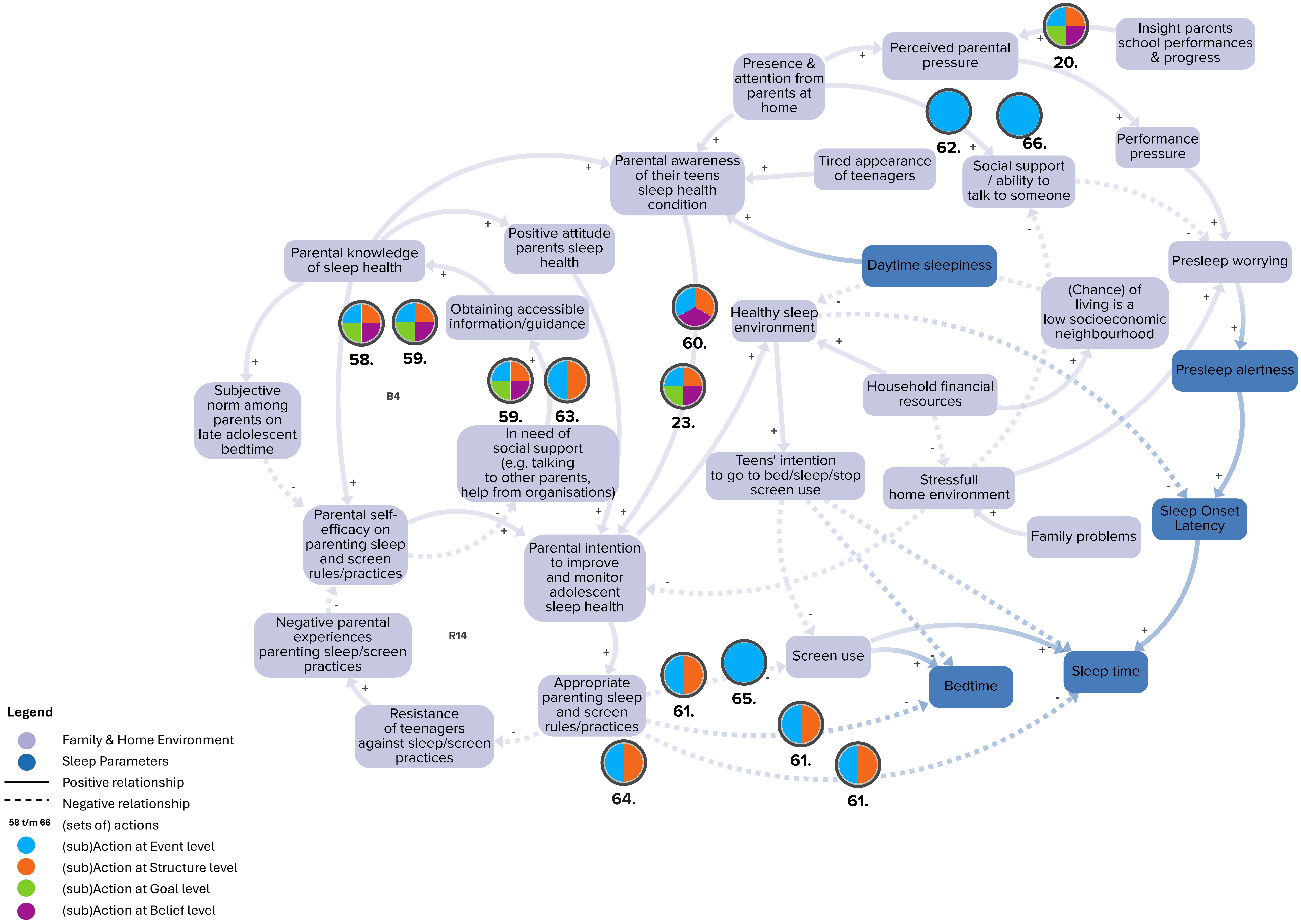

Insight parents school performances & progress
20.
Perceived parental pressure
Presence & attention from parents at home
62.
66.
Performance pressure
Tired appearance of teenagers
Social support / ability to talk to someone
Parental awareness of their teens sleep health condition
Positive attitude parents sleep health
Parental knowledge of sleep health
Daytime sleepiness
Presleep worrying
(Chance) of living is a low socioeconomic neighbourhood
Healthy sleep environment
60.
58.
59.
Obtaining accessible information/guidance
Presleep alertness
Household financial resources
Subjective norm among parents on late adolescent bedtime
B4
59.
63.
23.
In need of social support (e.g. talking to other parents, help from organisations)
Teens' intention to go to bed/sleep/stop screen use
Stressfull home environment
Sleep Onset Latency
Family problems
Parental self-efficacy on parenting sleep and screen rules/practices
Parental intention to improve and monitor adolescent sleep health
Negative parental experiences parenting sleep/screen practices
R14
Screen use
Sleep time
Bedtime
Appropriate parenting sleep and screen rules/practices
61.
65.
Resistance of teenagers against sleep/screen practices
61.
64.
61.
Legend
Family & Home Environment
Sleep Parameters
Positive relationship
Negative relationship
58 t/m 66
(sets of) actions
(sub)Action at Event level
(sub)Action at Structure level
(sub)Action at Goal level
(sub)Action at Belief level
