# Supplementary figures and images for: Developing a whole systems action plan promoting Dutch adolescents’ sleep health
Source: Int J Behav Nutr Phys Act. 2025 Mar 17;22:33. doi: 10.1186/s12966-025-01711-0 (PMC11917006; doi:10.1186/s12966-025-01711-0)

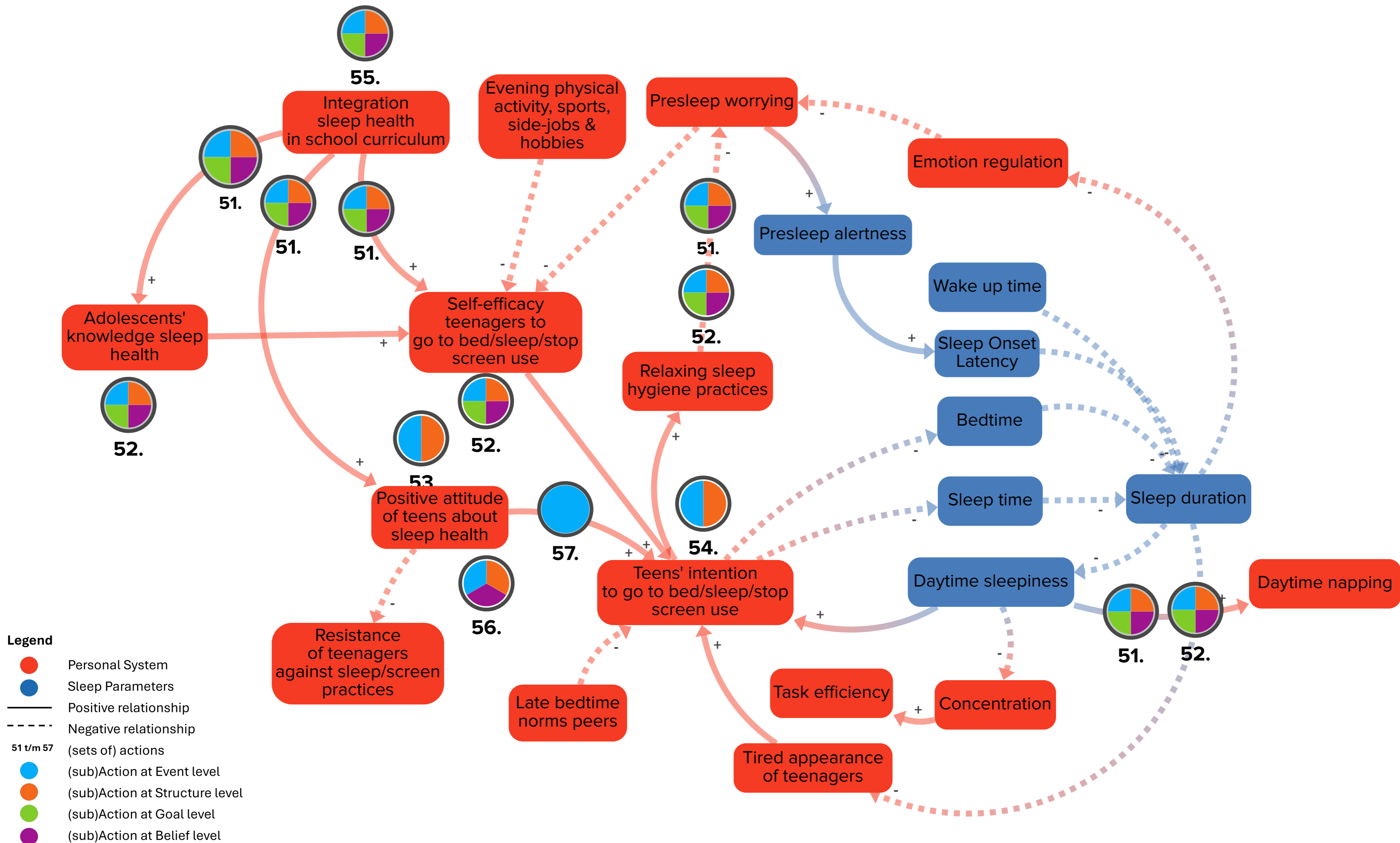

Supplement: Supplementary file 5 — Additional file 5. Causal loop diagram of the personal subsystem of adolescent sleep health, including all potential whole system action plan actions (adapted figure from Heemskerk et al. [ 6]. [file 12966_2025_1711_MOESM5_ESM.pdf]
